# The Reporting and Methodological Recommendations for Observational Studies Estimating the Effects of Deprescribing Medications (REMROSE‐D) ISPE‐Endorsed Guidance

**DOI:** 10.1002/pds.70255

**Published:** 2025-11-14

**Authors:** Kaleen N. Hayes, Joshua D. Niznik, Danijela Gnjidic, Frank Moriarty, Dimitri Bennett, Marie‐Laure Laroche, Denis Talbot, Matthew Alcusky, Maurizio Sessa, Antoinette B. Coe, Caroline Sirois, Andrew R. Zullo, Xiaojuan Li, Sri Harsha Chalasani, Jehath Syed, Mouna Sawan, Daniela C. Moga

**Affiliations:** ^1^ Department of Health Services, Policy, & Practice Brown University School of Public Health Providence Rhode Island USA; ^2^ Department of Epidemiology Brown University School of Public Health Providence Rhode Island USA; ^3^ Division of Geriatric Medicine and Center for Aging and Health University of North Carolina at Chapel Hill, School of Medicine Chapel Hill North Carolina USA; ^4^ Division of Pharmaceutical Outcomes and Policy University of North Carolina at Chapel Hill, Eshelman School of Pharmacy Chapel Hill North Carolina USA; ^5^ School of Pharmacy, Faculty of Medicine and Health The University of Sydney Sydney Australia; ^6^ School of Pharmacy and Biomolecular Sciences RCSI University of Medicine and Health Sciences Dublin Ireland; ^7^ Global Evidence and Outcomes, Takeda Development Center Americas, Inc Cambridge Massachusetts USA; ^8^ Centre of Pharmacovigilance and Pharmacoepidemiology, Department of Pharmacology‐Toxicology and Centre of Pharmacovigilance University Hospital of Limoges Limoges France; ^9^ UR 24134 Vie‐Santé, Faculty of Medicine University of Limoges, Campus Marcland Limoges France; ^10^ Department of Social and Preventive Medicine, Faculty of Medicine Université Laval Québec Canada; ^11^ Department of Population and Quantitative Health Sciences, UMass Chan Medical School Worcester Massachusetts USA; ^12^ Drug Safety Group, Department of Drug Design and Pharmacology University of Copenhagen Copenhagen Denmark; ^13^ Department of Clinical Pharmacy University of Michigan College of Pharmacy Ann Arbor Massachusetts USA; ^14^ Faculty of Pharmacy Université Laval Québec Canada; ^15^ Department of Population Medicine Harvard Medical School & Harvard Pilgrim Health Care Institute Boston Massachusetts USA; ^16^ Department of Pharmacy Practice, JSS College of Pharmacy JSS Academy of Higher Education & Research Mysuru India; ^17^ Department of Pharmaceutical Sciences, School of Health Sciences and Technology Dr. Vishwanath Karad MIT World Peace University Pune India; ^18^ Department of Pharmacy Practice and Science, College of Pharmacy; and Department of Epidemiology and Environmental Health, College of Public Health University of Kentucky Lexington Kentucky USA

**Keywords:** comparative effectiveness research, deprescribing, epidemiologic methods, geriatrics, inappropriate prescribing, pharmacoepidemiology, polypharmacy, real‐world evidence, research methodology, systematic review

## Abstract

**Purpose:**

Pharmacoepidemiologic studies on deprescribing are challenging to implement, yet little guidance exists on methods to avoid bias and minimum reporting for replicability and appraisal. We developed consensus recommendations for the methods and reporting of observational studies that aim to examine the effects of deprescribing.

**Methods:**

We formed candidate recommendations based on our prior systematic review that methodologically appraised observational studies on deprescribing. We then conducted a two‐round modified Delphi process with researchers working in deprescribing pharmacoepidemiology to refine, select, and reach consensus on recommendations for a checklist based on > 70% agreement of their importance. We termed this list the REMROSE‐D (Reporting and Methodological Recommendations for Observational Studies estimating the Effects of Deprescribing medications) guidance.

**Results:**

Twenty‐three candidate recommendations were presented to the Delphi panel. The round 1 survey was completed by 55 participants, and 18 of the 23 candidate recommendations were selected for inclusion. Five candidate recommendations without consensus plus two additional items suggested by participants were included in a round 2 survey of 25 deprescribing researchers. Five of these seven items garnered consensus for inclusion, and two were excluded. The final REMROSE‐D guidance contains 23 recommendations for the methods and reporting of observational research on deprescribing.

**Conclusion:**

To ensure rigor and reproducibility in observational studies of the effects of deprescribing, the REMROSE‐D guidance provides recommendations for important reporting and methods considerations, including time zero, precise definitions of deprescribing, addressing confounding by indication, and careful consideration of follow‐up to avoid immortal time bias.

**Summary**

- Observational studies of real‐world data are an efficient means to generate evidence on the effects of deprescribing medications, yet there is limited guidance for best practices in designing and reporting these studies.
- We leveraged a recent systematic review and implemented a modified Delphi process to obtain researcher consensus on the REMROSE‐D guidance—recommendations for reporting and best practices for addressing key methodological issues for observational studies on the effects of deprescribing.
- To adequately address potential biases, these recommendations focus on the reporting of detailed deprescribing treatment strategies, defining time zero, avoiding immortal time, and addressing confounding by indication.

## 1 Introduction

Deprescribing is the clinically supervised process of stopping, reducing, or tapering medications, with the ultimate goal of minimizing unnecessary polypharmacy, reducing complications from medication use, and improving quality of life [1]. Older adults are commonly the target population for deprescribing, as polypharmacy is often an inevitability for older adults and individuals with multiple chronic conditions; however, polypharmacy affects a wide range of populations [2, 3, 4] and thus deprescribing is relevant across the lifespan. A major barrier to deprescribing in routine practice is the limited availability of high‐quality, real‐world evidence for health‐related outcomes associated with deprescribing [5]. For example, prescribers and patients consistently note concerns regarding the unclear safety of stopping medications and potential rebound withdrawal effects as impediments to deprescribing [6, 7].

Although randomized controlled trials (RCTs) with treatment arms for continuing versus discontinuing treatment would be the gold standard for generating evidence on the effects of deprescribing, financial and logistical challenges, as well as barriers related to safety and acceptability, often preclude conducting such trials. Observational studies that leverage real‐world data (RWD) present an opportunity to circumvent these challenges, particularly for vulnerable groups who are a major population for deprescribing, but are difficult to enroll in trials (e.g., individuals living with dementia, medically complex children). These types of analyses also present an opportunity to more rapidly address current gaps in evidence by efficiently developing generalizable evidence in large, nationally representative populations. However, thoughtful consideration of the potential biases common to observational studies, particularly accounting for the inherent differences between individuals who continue versus discontinue treatment and time‐related biases, is necessary.

In the last decade, there has been an increasing number of published observational studies that leverage existing healthcare data to examine the effects of deprescribing medications on health‐related outcomes. To better understand the current landscape and limitations of deprescribing evidence generated using real‐world data, we recently conducted a systematic review wherein we identified and appraised data sources, study designs, and methodological approaches employed across 45 studies over the last 20 years [8]. Notable inconsistencies were observed regarding the measurement and reporting of deprescribing, exposure assessment windows, and time‐related biases.

In the absence of any established guidance or standardized reporting criteria for observational studies of deprescribing, in that review we identified several priority areas for improvement and emphasized the need for guidance on best practices for the design of these studies. Building on this work, the objective of the present study was to leverage this systematic review to develop a consensus‐based list of key reporting items and methodological considerations for observational studies that aim to estimate the effects of deprescribing medications. This article is endorsed by the International Society for Pharmacoepidemiology (ISPE).

## 2 Methods

### 2.1 Overview

**FIGURE 1:**
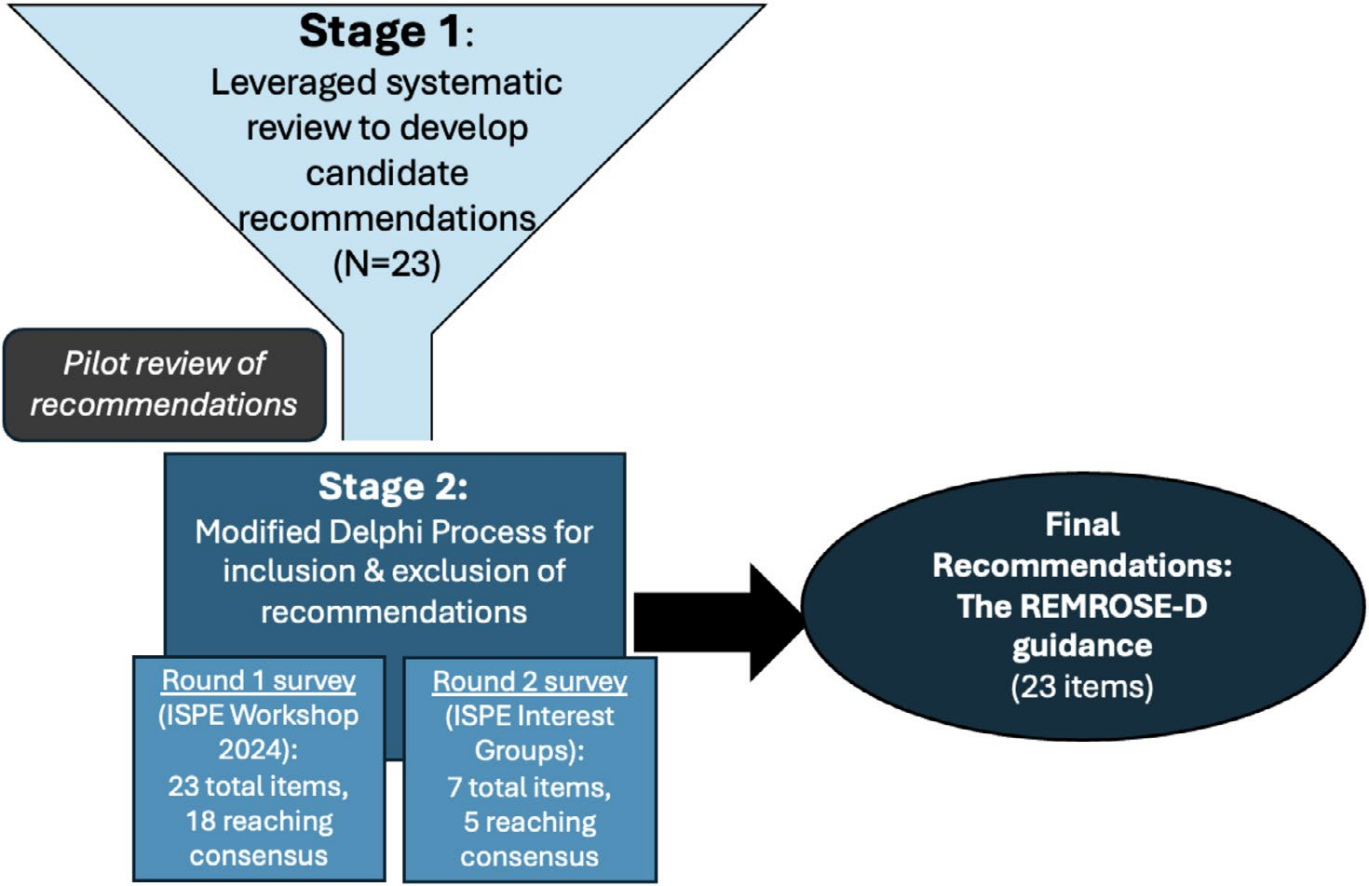
Graphical depiction of process to develop recommendations for observational studies of the effects of deprescribing.

We conducted a two‐stage process to develop, refine, and select recommendations for inclusion in the guidance, illustrated in Figure 1. In Stage 1, we leveraged the findings from our systematic review on methods in observational deprescribing research [8] to develop candidate recommendations related to reporting and methodological considerations with high importance to studies of deprescribing. In Stage 2, we implemented a modified Delphi process to establish broad consensus on inclusion, modification, and exclusion of the candidate recommendations in the final guidance. This work did not meet the definition of human subjects research and thus was deemed to be exempt from Institutional Review Board approval.

### 2.2 Stage 1: Formation of Candidate Recommendations

We previously conducted a systematic review of observational studies that examined the clinical effects of deprescribing using real‐world data [8]. This review was focused on studies examining deprescribing medications in older adults because they are the largest population targeted for deprescribing. In brief, we identified 45 eligible articles published between January 2000 and September 2023 and extracted 78 items on the study characteristics (e.g., year of publication, type of journal) and methods (e.g., exposure measurement, outcomes, and potential for time‐related biases) of each article. This systematic review identified variability in reporting and potential for major biases, primarily related to the measurement of deprescribing as an exposure and introducing immortal time related to exposure measurement. Results from this systematic review were leveraged to form candidate recommendations on reporting and methodological considerations for observational studies aiming to estimate the effects of deprescribing. Specifically, candidate recommendations were informed by aspects of reporting or methods with: (1) a high degree of missingness in reporting in the systematic review, (2) high variability between studies in the systematic review, or (3) they were specific or of critical importance to observational studies of deprescribing based on the consensus of coauthors of the systematic review (e.g., specifying a minimum medication‐free interval to define deprescribing as an exposure).

### 2.3 Stage 2: Modified Delphi Process

To refine and select final recommendations for the guidance, we conducted a two‐round modified Delphi process with international researchers in the field of deprescribing pharmacoepidemiology. We chose to allow broad participation of researchers with an interest in deprescribing in the consensus surveys rather than limiting it to selected experts in order to obtain diverse perspectives in considering both ideal and pragmatic study design and reporting decisions. In round 1, we engaged researchers working in deprescribing pharmacoepidemiology through a workshop on deprescribing research methods at the ISPE 2024 Annual Meeting in Berlin, Germany on August 26, 2024. To ensure that the audience had common foundational knowledge of the state of the literature and methods, results of the systematic review were presented (provided in Supporting Information S1) along with two other presentations related to deprescribing research methods. Then, in the same workshop session, a survey (Table S1) was distributed to participants. Participants were asked to rate the importance of each candidate item on a 5‐point Likert Scale (not important at all, somewhat unimportant, neutral: neither important/unimportant, somewhat important, or very important) or to vote if they were “unsure” about the importance of the item. The survey also contained a free‐text field where participants could add new items for consideration or other comments on the survey and items. We collected information about each participant's current country and career stage. After the survey was closed, a 20‐min discussion period was conducted to gather informal verbal feedback on the survey items and to discuss related concepts.

Items garnering > 70% agreement on importance (i.e., > 70% combined voted that an item was somewhat or very important) in round 1 of the survey were included in the final guidance. Items with > 70% agreement on unimportance (somewhat unimportant or not important at all) were excluded from further consideration. Though no optimal threshold for agreement has been established, we deemed 70% to be a sufficient threshold to recommend an item for all studies, while not limiting the checklist to only prioritize a subset of critical recommendations [9]. Prior studies related to real‐world evidence in medication studies have used this threshold [10]. We included all responses to each survey item when considering consensus, including those from participants who did not complete all items in the survey but did respond to that item. Thus, anyone who responded to an item was considered in the denominator of the proportion to determine consensus for that item. All other items that failed to achieve consensus for either inclusion or exclusion progressed to the second round of the survey. These items were reviewed by the research team for potential re‐wording or clarification based on the proportion of participants voting “unsure” as to the importance, free‐text feedback in the first round of the survey, and verbal feedback in the workshop discussion period. KH and JN also reviewed the free‐text feedback for potential additions to the survey for round 2. Coauthors were surveyed to vote on different rewording iterations of items and approve additions to the survey for round 2.

The round 2 survey only included items that did not garner consensus for inclusion or exclusion in the first round (with rewording, as applicable), plus additional items suggested in the first round. The round 2 survey was then sent to all members of the ISPE Geriatric Pharmacoepidemiology and Comparative Effectiveness Research Special Interest Groups (currently *n* = 556 total listed as of February 4, 2025) via the ISPE Exchange, an online community. This denominator of participants was designed to represent that of round 1 (researchers working in the field of deprescribing who are affiliated with ISPE), as we did not collect identifying information in the first round of the survey to directly re‐survey the same participants. The requirement for > 70% agreement on importance remained a requirement for inclusion; however, all other items that did not meet the requirement for inclusion were excluded after the second round of the survey.

## 3 Results

From the systematic review, we formulated 23 candidate recommendations. We then held a meeting with coauthors of the systematic review for discussion of the candidate recommendations before consideration by participants (Table S1). The first round of the consensus survey was completed in full by 55 participants, with 9 additional participants completing part of the survey. Tables S2a and S2b present round 1 participant characteristics. Forty percent were at the mid‐career or senior career stages. Others were early career (24%) or in training as doctoral students or postdoctoral fellows (36%). Most participants were from the US (36%), UK (11%), or Denmark (6.5%).

Of the 23 candidate items, 18 met the requirements for inclusion in the guidance based on survey results from round 1. None of the five remaining items met the criteria for exclusion and thus were considered for resurvey in the second round. One of these five items without consensus had a majority of respondents voting “unsure” and thus was reviewed for clarity and potential rewording prior to the second round of the survey. In addition, 19 unique responses were submitted for the free‐text question. Consensus from the authorship group on the rewording of one item from the initial survey and the addition of two new items generated from free‐text responses was established via a survey (*n* = 13 participants). A summary of responses to the first round of the survey, including rewording of the one item, is in Table S1, with free‐text responses and corresponding action items in Table S3.

Round 2 of the survey contained seven total items. Of these, five garnered consensus for inclusion and were included in the final recommendations. Two items still did not have consensus on importance and were excluded. Twenty‐five individuals participated in round 2, and results from round 2 of the survey are provided in Table S4. The final recommendation list (Table 1), termed the REMROSE‐D guidance (Reporting and Methodological Recommendations for Observational Studies estimating the Effects of Deprescribing medications), contains 23 total items. Minor wording modifications were made from the final consensus recommendations and the checklist for uniformity (e.g., “Investigators should follow a set of accepted general reporting guidelines…” was modified to “Follow a set of accepted general reporting guidelines…”). The REMROSE‐D guidance is divided into the following sections: (1) reporting and general study design, (2) deprescribing as an exposure, (3) assessment and outcomes, and (4) considerations for confounding. Table S5 provides a checklist that authors can leverage to show where in their manuscript they address each of the recommendations, where applicable and possible.

**TABLE 1:** The REMROSE‐D Guidance (Reporting and Methodological Recommendations for Observational Studies estimating the Effects of Deprescribing medications).

General reporting and study design	
Item 1	Follow a set of accepted general reporting guidelines for the specific type of observational study leveraged (e.g., International Society for Pharmacoepidemiology Good Pharmacoepidemiology Practice, Reporting of studies Conducted using Observational Routinely collected health Data statement for pharmacoepidemiology [RECORD‐PE], or the Strengthening The Reporting of Observational studies in Epidemiology [STROBE] guidelines).
Item 2	Present a study diagram that clearly identifies exposure, covariate, outcome, and eligibility assessment periods and the index date/time zero, as applicable.
Deprescribing as an exposure	
Item 3	Provide a detailed description of how exposure was assessed, including the data source (e.g., pharmacy claims), data transformations (e.g., data cleaning), how the last exposed date (e.g., last days supply) was determined, and any limitations of the data source to capture the most relevant form of deprescribing (e.g., commonly tapered or dose‐reduced medications), as applicable.
Item 4	Justify the decision to define deprescribing as completely stopping the medication (discontinuation), tapering the medication, or another definition in the context of the specific research question or clinical scenario.
Item 5	Discuss whether the definition of deprescribing was informed by clinical perspectives, including common prescribing practices and clinical practice guidelines.
Item 6	Discuss whether the definition of deprescribing was informed by pharmacokinetic considerations, including the duration of potential effects of the drug on outcomes after discontinuation.
Item 7	Discuss design and analytic choices implemented with exposure changes over time (e.g., restart of therapy after deprescribing, therapeutic substitution), including as‐treated vs. intention‐to‐treat analyses, censoring, and/or time‐varying exposure methods.
Item 8	Consider using similar definitions for deprescribing as prior studies, at least in secondary analyses, to enhance comparability of results.
Item 9	When applicable, avoid inducing immortal time (e.g., by misclassifying follow‐up time before meeting the minimum medication‐free interval into the “deprescribed” group or excluding it from analysis).
Item 10	Test the potential influence of the definition for deprescribing on study results through sensitivity analyses (e.g., changing required duration of change in use).
Assessment and outcomes	
Item 11	Discuss the time by which you would expect outcomes to occur relative to deprescribing (i.e., short‐term vs. long‐term outcomes).
Item 12	Include outcomes related to the negative potential consequences of deprescribing when possible.
Item 13	Include outcomes related to the potential benefits of deprescribing when possible.
Considerations for confounding	
Item 14	Discuss the comparator group in terms of potential for confounding, especially as it relates to differences between people with apparent deprescribing versus those who continue treatment.
Item 15	Select covariates based on the clinical and practical relevance to the selected drug class as permissible by the data source.
Item 16	Conduct sensitivity analyses regarding residual confounding, in particular confounding by indication.
Items 17–23	Consider the following covariates: Sociodemographic information, including age, race/ethnicity, sex, and geography. Clinical history and severity of the disease being studied (e.g., time since diabetes diagnosis) Co‐prescribed medications Comorbidities Prognosis and global assessments of comorbidity, frailty, or mortality risk Healthcare provider characteristics Relevant policies or clinical guidelines in place during the study period

## 4 Discussion

The REMROSE‐D guidance provides 23 recommendations for the reporting and methodological conduct of observational studies that aim to examine the effects of deprescribing. These recommendations were formed through a rigorous, sequential process beginning with a comprehensive ISPE‐endorsed systematic review of the methodology of studies of deprescribing and culminating with a multi‐round modified Delphi method to finalize the recommendations. The REMROSE‐D guidance aims to help researchers minimize major biases that are particularly harmful in deprescribing studies and ensure reporting beyond what is suggested for standard observational research so that studies can be appraised and replicated. However, despite the rigor of this guidance, estimating causal effects from observational data remains a challenging task [11] and these guidelines alone do not guarantee valid estimates. Finally, these recommendations are not meant to be inflexible “rules,” but rather important considerations that may have varying relevance depending on the specific research question and data source. In the following sections, we discuss the rationale and nuances for each recommendation.

### 4.1 Reporting and General Study Design

All observational studies should follow a reporting guideline appropriate for their study type. Examples include the ISPE Good Pharmacoepidemiology Practices (GPP) [12], the Reporting of studies Conducted using Observational Routinely collected health Data statement for pharmacoepidemiology (RECORD‐PE) [13], or the Strengthening The Reporting of Observational studies in Epidemiology (STROBE) guidelines [14]. Following a target trial emulation approach [15] can also serve as a reporting tool to reduce design‐induced biases. These tools establish the minimum standards for reporting observational studies and serve as a foundation for a rigorous, transparent, and reproducible study on deprescribing. Our additional reporting items are intended to build on this foundation. For example, while the ISPE GPP recommends providing “clear operational definitions of…exposures”, additional reporting items on the operational definitions of deprescribing as an exposure are provided as recommendations in this document.

Likewise, best pharmacoepidemiology practices include provision of a diagram that visually depicts study design choices for the exposure, outcomes, covariates, and other key methodological elements (e.g., censoring) [16]. Study diagrams are recommended by RECORD‐PE [13], but are often not part of other reporting guidelines that are used for nonpharmacoepidemiologic research. These diagrams are especially critical in deprescribing studies due to the complexities of establishing time‐zero and defining deprescribing exposures using real‐world data.

### 4.2 Deprescribing as an Exposure

Deprescribing is a complex exposure to ascertain in RWD, as it usually requires evidence of sustained absence or reduction of a medication after a baseline period of use. A first step is to provide detailed reporting of the data used to ascertain medication exposures (e.g., pharmacy claims with dispensing dates and days' supply, or prescription dates from EHR data with imputed durations of exposure). Reporting on data transformations, including data cleaning (e.g., extracting days supply values from dispensings or doses from administrations) should also be described either in the manuscript, supplement, or through shared statistical code, as recommended where possible for pharmacoepidemiologic studies [17].

An explicit definition of deprescribing is critical to appraise and potentially replicate a study. This definition should include a detailed description of the deprescribing exposure (e.g., gap in use > 90 days, first prescription for a lower dose). Methods used to determine days exposed and unexposed to medications are a key piece of exposure reporting. For example, if pharmacy dispensing claims were used, investigators should report whether a grace period (allowable gap) was added to the last days supply. The optimal allowable gap depends on the clinical context and research question; researchers should weigh the balance of misclassifying temporary gaps in treatment (e.g., 1–2 inadvertent missed prescription fills) as deprescribing with shorter gaps and reducing sample size and follow‐up time with longer gaps. Using a longer allowable gap may also be appropriate when using orders data in EHR rather than claims due to a lack of data on exact prescription dispensing and refills. Regardless, researchers ideally would test the impact of using different allowable gap lengths in a sensitivity analysis (Box 1). Investigators should also consider, when relevant, how periods with missing medication use were handled, such as hospitalizations and postacute care periods. These key details are important for deprescribing medications not only to understand the operational definition of deprescribing, but also for studies with time‐varying exposures and as‐treated effects that consider changes in medication use continually over follow‐up. Of note, however, is that estimation of time‐varying exposure effects may require the use of advanced analytic methods for causal inference, such as marginal structural models [18].

BOX 1 **Demonstration of Misclassification Bias.**

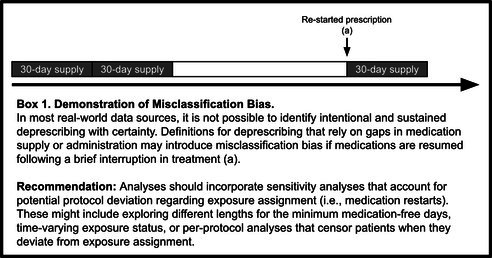

Next, acknowledgment of limitations of the data source to capture the most relevant form(s) of deprescribing should also be addressed. For example, some medications, like selective serotonin reuptake inhibitors, cannot be abruptly stopped and thus may require long periods of follow‐up to determine discontinuation. Many data sources lack information on directions for use or tapering instructions that are important for measuring deprescribing in this case. Instructions for use may also contain key information necessary for understanding deprescribing—for example, measuring a reduction in insulin dose or schedules. Medications for conditions with treatment targets (e.g., diabetes, hypertension) may be deprescribed via reducing the intensity (e.g., dose, frequency, or number of medications) rather than complete discontinuation. Investigators should justify the type of deprescribing studied based on the clinical question of interest and data availability.

Finally, even with thoughtful study design and intentional sensitivity analyses, measurement of deprescribing in real‐world data usually relies on an observed change in medication use without the ability to distinguish between intentional deprescribing and other reasons for medication cessation. For example, medication cessation can result from circumstances other than deprescribing (e.g., an individual independently decides to or inadvertently discontinues the medication) [19]. As such, observing a restart of therapy after discontinuation is not uncommon (Box 1), and the required duration of sustained change in medication use can greatly modify the number of people classified as “deprescribed.” [20] All studies should report the type of analysis (e.g., modified intention to treat [people could not switch out of the deprescribing group], as‐treated analysis [time‐varying exposures, censoring upon re‐start of a medication, etc.]), and investigators should explicitly report what was done in the event that someone restarted therapy after discontinuation. Finally, regardless of whether time‐varying exposures are used or not, the complexity of measuring deprescribing in real‐world data may also create opportunities for inducing immortal time. Specifically, in most traditional cohort studies, misclassification of follow‐up time as being “deprescribing‐exposed” before meeting the minimum period of medication‐free days will lead to incorrect estimation of the effect of deprescribing by inducing immortal time (Box 2). Additional references on immortal time bias, and advanced methods to address it like clone‐censor‐weighting in target trial emulation [21], are available outside of this paper [22, 23, 24, 25].

BOX 2 **Demonstration of Immortal Time.**

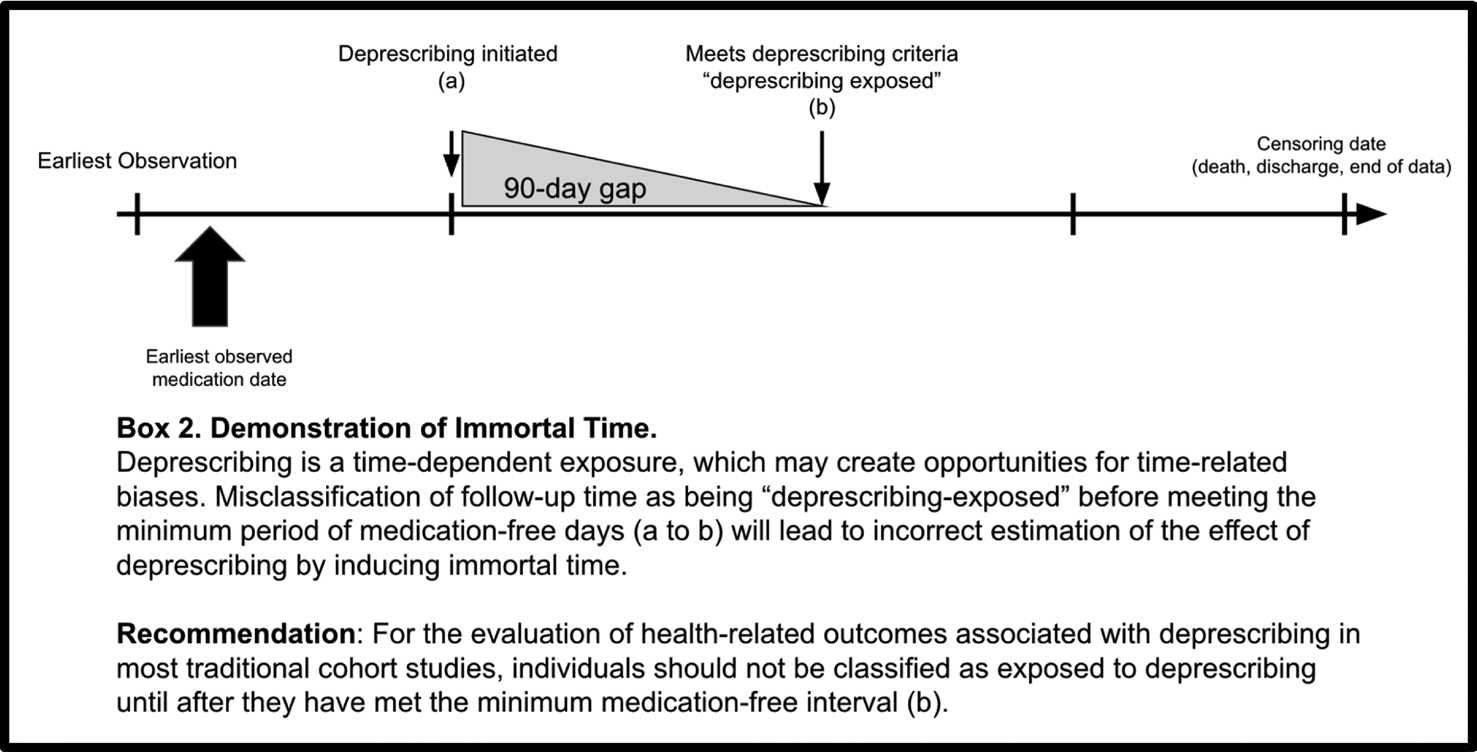

### 4.3 Assessment and Outcomes

The ultimate goal of observational studies examining the effects of deprescribing is to generate evidence that providers, patients, and caregivers can use to weigh the potential benefits and harms of continuing versus discontinuing medications. Further, evidence on deprescribing can also be of use to policymakers and payers in informing public health policies or insurance coverage aimed at optimizing medication use. In qualitative studies, both providers and patients are consistently concerned about the potential harm or rebound effects that may result from deprescribing [6, 7]. Following suit, clinical and observational studies examining the effects of deprescribing using real‐world data have also been largely focused on evaluating potential harm or rebound effects [5, 8]. However, evidence supporting the potential beneficial effects of deprescribing (e.g., reduced medication burden, reduced likelihood of medication‐related adverse effects) has the potential to provide a more convincing argument in favor of deprescribing, particularly when studies indicate there is no evidence of increased harms from deprescribing.

Evaluating the association of deprescribing with a hypothetical reduction in serious medication‐specific adverse events can be operationalized in real‐world data by examining diagnoses on hospitalization and emergency visit records [11]. However, person‐centered outcomes such as quality of life and medication burden may be more difficult to measure. Finally, investigators should consider the plausible length of time that an outcome could be attributed to the exposure of deprescribing based on the pharmacokinetics of the medication, short‐ versus long‐term onset, and clinical reasoning. Many outcomes (e.g., falls, medication burden, medication‐related adverse events) could reasonably have a rapid onset after deprescribing. However, lagged outcome measurement or other methods to adjust exposure and follow‐up time may be appropriate in some studies. For example, due to the long half‐lives of bisphosphonates, fractures occurring shortly after medication cessation are unlikely to be caused by the discontinuation of the medication.

### 4.4 Considerations for Confounding

Adequate consideration of potential confounders is critical in examinations of deprescribing with health‐related outcomes. First, careful consideration of the comparator group (i.e., the group that will be compared to those being deprescribed) is important. Comparator groups in deprescribing research often comprise patients who continue treatment. Confounding by indication is a particular concern for deprescribing research, as the reason for discontinuation is often unknown, and individuals for whom deprescribing is most likely may also have an increased risk of adverse health outcomes and mortality. For example, those in whom statins are discontinued because of limited life expectancy may also be at high risk of cardiovascular events.

Thus, the selection of the comparator group should consider how patients in the deprescribing exposure group may differ from other patient populations. When the comparator group consists of individuals who continue the same treatment, investigators may consider using time‐distribution matching techniques to ensure that the start of follow‐up is balanced across groups. This may be particularly relevant when the study design is grounded in the context of healthcare episodes (e.g., nursing home stays, following hospice enrollment) during which deprescribing may occur at any point but factors such as prognosis and risk for negative events shift over time. Alternatively, investigators might consider using an active comparator design. An active comparator design traditionally refers to comparing the effects of “Drug A” versus “Drug B,” rather than comparing “Drug A” versus “no medication use,” given the potential for unmeasured confounding between users and nonusers of medications [26]. In the context of deprescribing studies, an active comparator would be a population with similar clinical characteristics, prognosis, and patterns of healthcare utilization to those undergoing the deprescribing exposure of interest. For example, comparing outcomes between those with two forms of deprescribing (e.g., dose reduction versus complete discontinuation, deprescribing of drugs from different medication classes or with different indications) may serve as a form of active comparison to partially address confounding by indication, since both groups had a change in medication use. Finally, in situations where deprescribing practice patterns vary between individual providers or between facilities (e.g., recently admitted nursing home residents who discontinue versus continue a medication), there may be opportunities to apply instrumental variable methods to estimate the effects of deprescribing. Examples might include nursing home facility prescribing preference, which has been demonstrated to be a suitable instrumental variable for comparative effectiveness studies [27]. However, making sure a candidate instrument is truly an instrumental variable in a given study remains challenging [28]. Sensitivity analyses that quantify the impact of residual confounding are also a key consideration. Potential methods are varied and can include restricted subgroup analyses, use of negative control exposures or outcomes [29], the *E*‐value [30] or “array approach” [31] for assessing the impact of residual confounders, and quantitative bias analyses [32].

Secondly, studies should account for potential confounding analytically through a priori consideration of a host of important covariates identified through consensus. We recommend prespecifying these variables even when implementing data driven approaches like high‐dimensional propensity scores [33]. Several frameworks [34, 35, 36] have been developed that identify potential intrapersonal, interpersonal, and system‐level influences on deprescribing as a health behavior that can guide covariate selection to address potential confounding and examine key subgroups that may be at risk of greater benefits or harms from deprescribing. One such framework by Thorpe [37] and colleagues provides an in‐depth breakdown of these influences into constructs along with suggestions for measurement in administrative healthcare data sources. The results from our consensus evaluation generally agreed with this framework, which allows for flexibility depending on the drug class being studied. Our recent systematic review also provides support for the importance of potential confounders across these constructs, as more than half of published observational studies on deprescribing incorporated sociodemographics, clinical history and disease severity, co‐prescribed medications, comorbidities, and prognosis. Particularly salient confounders for deprescribing research relate to attributes of medication and clinical conditions, like the time since drug initiation (i.e., time on treatment) and duration of illness for the treated indication, which may serve as proxies of disease progression and the potential for benefit from the treatment (e.g., a short duration of statin use is unlikely to have enduring benefit after discontinuation). Finally, although participants determined that both healthcare provider characteristics and relevant policies/guidelines are important to consider as covariates in studies, fewer than 25% of studies in the systematic review considered these variables. Some policy‐related variables, such as policies governing medication administration and review across nursing home chains and prescription medication plan formularies, may vary substantially between individuals and thus be feasible to include in a model. Other variables may not be appropriate as covariates in a model if they apply to all individuals in a study population, but are likely critically important for study design and interpretation of results. As a simple example, new studies on the effects of statin deprescribing in a US population may want to consider only examining data after 2013, when the American College of Cardiology and American Heart Association guidelines were updated to expand the indications for statins [38].

## 5 Strengths and Limitations

This study and the REMROSE‐D guidance have several important strengths. First, we leveraged the largest and most rigorous systematic review to date of observational studies on the effects of deprescribing to form candidate recommendations. These candidate recommendations were developed empirically on the basis of results of the review, including considerations of missingness, variability, and consensus‐determined importance. Candidate recommendations were then carefully considered and modified based on multiple rounds of consensus surveys with over 70 participants working in deprescribing pharmacoepidemiology from 20 different countries. There are limitations to this work, however. Firstly, while we believe that the inclusion of any researcher with an interest in deprescribing in the consensus surveys, rather than only selected experts, helps to make these recommendations broad and pragmatic, we also recognize the limitation that not all participants may have been highly experienced in the conduct of observational research in deprescribing. Responses may also have been influenced by response bias, including acquiescence bias (i.e., each item was deemed very important simply by nature of being considered) and personal bias (e.g., a potential covariate may have been deemed unimportant if a participant works primarily with data that does not include that information). Next, the survey may have an over‐representation of certain perspectives, particularly those from North America and Western Europe and English speakers, despite our aim for broad inclusion and attempt to incorporate both in‐person and virtual opportunities to participate. Due to an inability to collect participant contact information from round 1, we also did not recruit the same participants from the round 1 survey to participants in round 2, which may have introduced heterogeneity in perspectives between the rounds. We believe this heterogeneity would be limited due to engaging participants affiliated with ISPE and deprescribing research in both rounds, likely resulting in overlap in participants. However, we are unable to quantify differences in characteristics between participants in round 1 vs. round 2.

These recommendations add to standard reporting and methodological considerations of observational studies, with the goals of transparency and reproducibility. However, these recommendations cannot be guaranteed to be comprehensive and may need to be updated over time as research questions, methodological approaches, and data sources evolve. Moreover, these recommendations are meant to be a guide and might not be applicable to all studies; the REMROSE‐D checklist allows for explanation when a recommendation is not applicable or possible with a given data source or research question.

## 6 Conclusions

Observational studies that aim to establish the effects of deprescribing on clinical outcomes are complex. To ensure rigor and reproducibility, they require detailed reporting, particularly regarding how deprescribing treatment strategies are defined. Moreover, appropriate methods to define time zero, avoid immortal time, and address confounding by indication are imperative to reduce the potential for bias in deprescribing pharmacoepidemiology. The recommendations formed through multiple rounds of consensus and discussion in the REMROSE‐D guidance represent an important first step to address these challenges. Future research should evaluate whether adoption of these recommendations improves study quality, reduces the potential for bias, and facilitates decision‐making by investigators conducting observational studies of deprescribing.

### 6.1 Plain Language Summary

Using real‐world data to understand the benefits and harms of stopping or reducing medications (i.e., deprescribing medications) is a rapidly growing field of research. However, there has been limited guidance on how to design these studies in ways that reduce important biases. In a previous systematic review, we described key study design elements of published research that used real‐world data to examine the effect of deprescribing medications on patient outcomes. In this study, we asked researchers in the field to rate the importance of these elements for designing, conducting, and reporting observational research examining the effect of deprescribing medications on health‐related outcomes. Participants emphasized the importance of clearly defining how they measured deprescribing in their data, reporting periods in which they observed exposures and outcomes, and how they addressed potential clinical differences between people stopping versus continuing medications in real‐world data (known as confounding). By combining our systematic review with broad consensus, we developed the Reporting and Methodological Recommendations for Observational Studies estimating the Effects of Deprescribing medications (REMROSE‐D) guidance that researchers can use to enhance transparency and reduce bias in studies estimating the effects of deprescribing.

## Ethics Statement

This work did not meet the definition of human subjects research and thus was deemed to be exempt from Institutional Review Board approval.

## Conflicts of Interest

K.N.H. has received grant funding paid directly to Brown University for investigator‐initiated research from Sanofi, Genentech, and GlaxoSmithKline for research on influenza vaccination in nursing homes, influenza outbreak control, and shingles vaccination in nursing homes, respectively. D.B. is an employee of Takeda. X.L. has received support via funding from the NIH/NIA (K01AG073651). A.B.C. has received support via funding from the NIH/NIA (K08AG071856). D.T. was supported by a research career award from the Fonds de recherche du Québec–Santé. All other authors declare no conflicts of interest.

## Supporting information


**Data S1:** pds70255‐sup‐0001‐Supinfo.docx.

## References

[1] E. Reeve , D. Gnjidic , J. Long , and S. Hilmer , “A Systematic Review of the Emerging Definition of “Deprescribing” With Network Analysis: Implications for Future Research and Clinical Practice,” British Journal of Clinical Pharmacology 80, no. 6 (2015): 1254–1268, 10.1111/bcp.12732. 27006985 PMC4693477

[2] J. Guillot , S. Maumus‐Robert , A. Pariente , and J. Bezin , “Chronic Polypharmacy at all Age: A Population‐Based Drug Utilization Study,” Fundamental & Clinical Pharmacology 36, no. 2 (2022): 405–413, 10.1111/fcp.12727. 34506043

[3] J. Guillot , C. T. Rentsch , K. S. Gordon , A. C. Justice , and J. Bezin , “Potentially Inappropriate Medication Use by Level of Polypharmacy Among US Veterans 49–64 and 65–70 Years Old,” Pharmacoepidemiology and Drug Safety 31, no. 10 (2022): 1056–1074, 10.1002/pds.5506. 35780391 PMC9464694

[4] N. Golchin , H. Johnson , P. M. Bakaki , et al., “Outcome Measures in Pediatric Polypharmacy Research: A Scoping Review,” Drugs & Therapy Perspectives 35, no. 9 (2019): 447–458, 10.1007/s40267-019-00650-8. 32256042 PMC7123381

[5] E. Reeve , W. Thompson , and B. Farrell , “Deprescribing: A Narrative Review of the Evidence and Practical Recommendations for Recognizing Opportunities and Taking Action,” European Journal of Internal Medicine 38 (2017): 3–11, 10.1016/j.ejim.2016.12.021. 28063660

[6] J. Bolt , R. Abdoulrezzak , and C. Inglis , “Barriers and Enablers to Deprescribing of Older Adults and Their Caregivers: A Systematic Review and Meta‐Synthesis,” European Geriatric Medicine 14, no. 6 (2023): 1211–1222, 10.1007/s41999-023-00879-7. 37874489

[7] A. Burghle , C. Lundby , J. Ryg , et al., “Attitudes Towards Deprescribing Among Older Adults With Limited Life Expectancy and Their Relatives: A Systematic Review,” Drugs & Aging 37, no. 7 (2020): 503–520, 10.1007/s40266-020-00774-x. 32537682

[8] K. N. Hayes , J. D. Niznik , D. Gnjidic , et al., “Evaluation of Real‐World Evidence to Assess Health Outcomes Related to Deprescribing Medications in Older Adults: An International Society for Pharmacoepidemiology‐Endorsed Systematic Review of Methodology,” American Journal of Epidemiology 194 (2024): 2431–2439, 10.1093/aje/kwae425. PMC12342872 39572376

[9] S. Drumm , C. Bradley , and F. Moriarty , “‘More of an Art Than a Science’? The Development, Design and Mechanics of the Delphi Technique,” Research in Social & Administrative Pharmacy 18, no. 1 (2022): 2230–2236, 10.1016/j.sapharm.2021.06.027. 34244078

[10] I. M. Kronish , C. T. Thorpe , and C. I. Voils , “Measuring the Multiple Domains of Medication Nonadherence: Findings From a Delphi Survey of Adherence Experts,” Translational Behavioral Medicine 11, no. 1 (2021): 104–113, 10.1093/tbm/ibz133. 31580451 PMC7877304

[11] X. Li , E. A. Bayliss , M. A. Brookhart , and M. L. Maciejewski , “Assessing Causality in Deprescribing Studies: A Focus on Adverse Drug Events and Adverse Drug Withdrawal Events,” Journal of the American Geriatrics Society 73, no. 3 (2025): 697–706, 10.1111/jgs.19241. 39446059 PMC11908924

[12] Public Policy Committee, International Society of Pharmacoepidemiology , “Guidelines for Good Pharmacoepidemiology Practice (GPP),” Pharmacoepidemiology and Drug Safety 25, no. 1 (2016): 2–10, 10.1002/pds.3891. 26537534

[13] S. M. Langan , S. A. Schmidt , K. Wing , et al., “The Reporting of Studies Conducted Using Observational Routinely Collected Health Data Statement for Pharmacoepidemiology (RECORD‐PE),” BMJ (Clinical Research Ed.) 363 (2018): k3532, 10.1136/bmj.k3532. PMC6234471 30429167

[14] E. von Elm , D. G. Altman , M. Egger , et al., “The Strengthening the Reporting of Observational Studies in Epidemiology (STROBE) Statement: Guidelines for Reporting Observational Studies,” Annals of Internal Medicine 147, no. 8 (2007): 573–577, 10.7326/0003-4819-147-8-200710160-00010. 17938396

[15] M. A. Hernán , W. Wang , and D. E. Leaf , “Target Trial Emulation: A Framework for Causal Inference From Observational Data,” JAMA 328, no. 24 (2022): 2446–2447, 10.1001/jama.2022.21383. 36508210

[16] S. Schneeweiss , J. A. Rassen , J. S. Brown , et al., “Graphical Depiction of Longitudinal Study Designs in Health Care Databases,” Annals of Internal Medicine 170, no. 6 (2019): 398–406, 10.7326/M18-3079. 30856654

[17] J. Tazare , S. V. Wang , R. Gini , et al., “Sharing Is Caring? International Society for Pharmacoepidemiology Review and Recommendations for Sharing Programming Code,” Pharmacoepidemiology and Drug Safety 33, no. 9 (2024): e5856, 10.1002/pds.5856. 39233394

[18] J. M. Robins , M. A. Hernán , and B. Brumback , “Marginal Structural Models and Causal Inference in Epidemiology,” Epidemiology 11, no. 5 (2000): 550–560, 10.1097/00001648-200009000-00011. 10955408

[19] E. A. Bayliss , G. K. Goodrich , J. C. Barrow , et al., “Discontinuation Categories Underlying Gaps in Dispensing for Six Medication Groups,” Pharmacoepidemiology and Drug Safety 34, no. 4 (2025): e70142, 10.1002/pds.70142. 40197856 PMC12380114

[20] J. D. Niznik , S. Shmuel , V. Pate , et al., “Validating Claims‐Based Definitions for Deprescribing: Bridging the Gap Between Clinical and Administrative Data,” Pharmacoepidemiology and Drug Safety 33, no. 4 (2024): e5784, 10.1002/pds.5784. 38556843 PMC11145562

[21] C. E. Gaber , K. A. Hanson , S. Kim , J. L. Lund , T. A. Lee , and E. J. Murray , “The Clone‐Censor‐Weight Method in Pharmacoepidemiologic Research: Foundations and Methodological Implementation,” Current Epidemiology Reports 11, no. 3 (2024): 164–174, 10.1007/s40471-024-00346-2.

[22] S. Suissa , “Immortal Time Bias in Pharmaco‐Epidemiology,” American Journal of Epidemiology 167, no. 4 (2008): 492–499, 10.1093/aje/kwm324. 18056625

[23] S. Suissa , “Immortal Time Bias in Observational Studies of Drug Effects,” Pharmacoepidemiology and Drug Safety 16, no. 3 (2007): 241–249, 10.1002/pds.1357. 17252614

[24] L. E. Lévesque , J. A. Hanley , A. Kezouh , and S. Suissa , “Problem of Immortal Time Bias in Cohort Studies: Example Using Statins for Preventing Progression of Diabetes,” BMJ 340 (2010): b5087, 10.1136/bmj.b5087. 20228141

[25] M. A. Hernán , J. A. C. Sterne , J. P. T. Higgins , I. Shrier , and S. Hernández‐Díaz , “A Structural Description of Biases That Generate Immortal Time,” Epidemiology 36, no. 1 (2025): 107–114, 10.1097/EDE.0000000000001808. 39494894 PMC11598638

[26] K. Yoshida , D. H. Solomon , and S. C. Kim , “Active‐Comparator Design and New‐User Design in Observational Studies,” Nature Reviews Rheumatology 11, no. 7 (2015): 437–441, 10.1038/nrrheum.2015.30. 25800216 PMC4486631

[27] K. F. Huybrechts , T. Gerhard , J. M. Franklin , R. Levin , S. Crystal , and S. Schneeweiss , “Instrumental Variable Applications Using Nursing Home Prescribing Preferences in Comparative Effectiveness Research,” Pharmacoepidemiology and Drug Safety 23, no. 8 (2014): 830–838, 10.1002/pds.3611. 24664805 PMC4116440

[28] E. P. Martens , W. R. Pestman , A. De Boer , S. V. Belitser , and O. H. Klungel , “Instrumental Variables: Application and Limitations,” Epidemiology 17, no. 3 (2006): 260–267, 10.1097/01.ede.0000215160.88317.cb. 16617274

[29] M. Lipsitch , E. Tchetgen Tchetgen , and T. Cohen , “Negative Controls: A Tool for Detecting Confounding and Bias in Observational Studies,” Epidemiology 21, no. 3 (2010): 383–388, 10.1097/EDE.0b013e3181d61eeb. 20335814 PMC3053408

[30] T. J. VanderWeele and P. Ding , “Sensitivity Analysis in Observational Research: Introducing the E‐Value,” Annals of Internal Medicine 167, no. 4 (2017): 268–274, 10.7326/M16-2607. 28693043

[31] S. Schneeweiss , “Sensitivity Analysis and External Adjustment for Unmeasured Confounders in Epidemiologic Database Studies of Therapeutics,” Pharmacoepidemiology and Drug Safety 15, no. 5 (2006): 291–303, 10.1002/pds.1200. 16447304

[32] T. L. Lash , M. P. Fox , R. F. MacLehose , G. Maldonado , L. C. McCandless , and S. Greenland , “Good Practices for Quantitative Bias Analysis,” International Journal of Epidemiology 43, no. 6 (2014): 1969–1985, 10.1093/ije/dyu149. 25080530

[33] J. A. Rassen , P. Blin , S. Kloss , et al., “High‐Dimensional Propensity Scores for Empirical Covariate Selection in Secondary Database Studies: Planning, Implementation, and Reporting,” Pharmacoepidemiology and Drug Safety 32, no. 2 (2023): 93–106, 10.1002/pds.5566. 36349471 PMC10099872

[34] A. Todd , J. Jansen , J. Colvin , and A. J. McLachlan , “The Deprescribing Rainbow: A Conceptual Framework Highlighting the Importance of Patient Context When Stopping Medication in Older People,” BMC Geriatrics 18, no. 1 (2018): 295, 10.1186/s12877-018-0978-x. 30497404 PMC6267905

[35] K. T. Bain , H. M. Holmes , M. H. Beers , V. Maio , S. M. Handler , and S. G. Pauker , “Discontinuing Medications: A Novel Approach for Revising the Prescribing Stage of the Medication‐Use Process,” Journal of the American Geriatrics Society 56, no. 10 (2008): 1946–1952, 10.1111/j.1532-5415.2008.01916.x. 18771457 PMC3119470

[36] A. Linsky , W. F. Gellad , J. A. Linder , and M. W. Friedberg , “Advancing the Science of Deprescribing: A Novel Comprehensive Conceptual Framework,” Journal of the American Geriatrics Society 67, no. 10 (2019): 2018–2022, 10.1111/jgs.16136. 31430394 PMC6800794

[37] C. Thorpe , J. Niznik , and A. Li , “Deprescribing Research in Nursing Home Residents Using Routinely Collected Healthcare Data: A Conceptual Framework,” BMC Geriatrics 23, no. 1 (2023): 469, 10.1186/s12877-023-04194-5. 37542226 PMC10401751

[38] N. J. Stone , J. G. Robinson , A. H. Lichtenstein , et al., “2013 ACC/AHA Guideline on the Treatment of Blood Cholesterol to Reduce Atherosclerotic Cardiovascular Risk in Adults,” Journal of the American College of Cardiology 63, no. 25 (2014): 2889–2934, 10.1016/j.jacc.2013.11.002. 24239923

