# Supplementary material for: The Reporting and Methodological Recommendations for Observational Studies Estimating the Effects of Deprescribing Medications (REMROSE‐D) ISPE‐Endorsed Guidance
Source: Pharmacoepidemiol Drug Saf. 2025 Nov 14;34(11):e70255. doi: 10.1002/pds.70255 (PMC12617387; doi:10.1002/pds.70255)

**SUPPLEMENTARY MATERIALS**

*REporting and Methodological Recommendations for Observational Studies Estimating the effects of Deprescribing medications: the* International Society for Pharmacoepidemiology-endorsed *REMROSE-D guidance*

*Hayes, Niznik, Gnjidic, et al.*

**Table S1.** Survey items and responses (Round 1).

**Table S2a.** Distribution of geographic location of round 1 participants (N=61).

**Table S2b.** Distribution of career stage of round 1 participants (N=66).

**Table S3.** Free text responses from Round 1 of the survey and actions taken.

**Table S4.** Survey items and responses (Round 2).

**Table S5**. Reporting and Methods Checklist for Observational Studies on the Effects of Deprescribing.

**Supplemental Material 1.** Slides from presentation delivered before Round 1 survey.

**Table S1.** Survey items and responses (Round 1).

| **Item Number** | **Item** | **Not Important or Somewhat Unimportant (N, % of total responses for that item)** | **Neither Important nor Unimportant (N, % of total responses for that item)** | **Somewhat Important or Very Important (N, % of total responses for that item)** | **Unsure** | **Result** |
| --- | --- | --- | --- | --- | --- | --- |
| 1 | Investigators should follow a set of accepted general reporting guidelines for the specific type of observational study leveraged (e.g., STROBE or EQUATOR guidelines). | 1 (2%) | 1 (2%) | 62 (97%) | 0 (0%) | Include |
| 2 | Investigators should present a study diagram that clearly identifies exposure,  covariate, outcome, and eligibility assessment periods and the index date/time zero, as applicable. | 0 (0%) | 1 (2%) | 60 (97%) | 1 (2%) | Include |
| 3 | Investigators should consider the comparator group carefully due to the potential for confounding between people who discontinue a medication vs. continue treatment. | 0 (0%) | 1 (2%) | 60 (96%) | 2 (3%) | Include |
| 4 | Investigators should precisely report how exposure was assessed, including the  data source, data transformations, and how the last exposed date\was determined. | 0 (0%) | 1 (2%) | 41 (98%) | 0 (0%) | Include |
| 5 | Investigators should justify the decision to define deprescribing as completely  stopping the medication, tapering the medication, or another definition in the context of the specific research question or clinical scenario. | 0 (0%) | 0 (0%) | 59 (99%) | 1 (2%) | Include |
| 6 | The definition of deprescribing should be informed by: Consideration of the time  by which you would expect outcomes to occur relative to deprescribing (i.e.,  short-term vs. long-term outcomes). | 4 (6%) | 3 (5%) | 47 (78%) | 6 (10%) | Include |
| 7 | The definition of deprescribing should be informed by: Clinical perspectives,  including common prescribing practices and clinical practice guidelines. | 0 (0%) | 0 (0%) | 58 (98%) | 1 (2%) | Include |
| 8 | The definition of deprescribing should be informed by: Pharmacokinetic  considerations, including the duration of potential effects of the drug outcomes after discontinuation. | 4 (7%) | 3 (5%) | 49 (83%) | 3 (5%) | Include |
| 9 | The potential influence of the definition for deprescribing on study results should  be tested through sensitivity analyses (e.g., changing required duration of change in use). | 0 (0%) | 4 (7%) | 53 (90%) | 2 (3%) | Include |
| 10 | Investigators should consider using similar definitions for deprescribing as prior  studies, at least in secondary analyses, to enhance comparability of results. | 5 (9%) | 8 (14%) | 43 (74%) | 2 (3%) | Include |
| 11 | When applicable, investigators should avoid classifying follow-up time before meeting the minimum medication-free interval as being "deprescribing-exposed." | 2 (4%) | 5 (9%) | 31 (55%) | 19 (33%) | Reword and Resurvey  Accepted Rewording for Round 2: *“When applicable, investigators should avoid inducing immortal time (e.g., by misclassifying follow-up time before meeting the minimum medication-free interval into the "deprescribed" group or excluding it from analysis).”* |
| 12 | Investigators should include outcomes related to the negative potential consequences of deprescribing when possible. | 1 (2%) | 4 (7%) | 53 (91%) | 0 (0%) | Include |
| 13 | Investigators should include outcomes related to the potential benefits of deprescribing when possible. | 2 (4%) | 7 (12%) | 48 (85%) | 0 (0%) | Include |
| 14 | Covariates examined are specific to the research question and data source but should also be selected based on clinical and practical relevance to the selected  drug class. | 0 (0%) | 1 (2%) | 55 (96%) | 1 (2%) | Include |
| 15 | Investigators should consider including the following covariate in studies of deprescribing: Sociodemographic information, including age, race/ethnicity, sex,  and geography. | 1 (2%) | 5 (9%) | 51 (90%) | 0 (0%) | Include |
| 16 | Investigators should consider including the following covariate in studies of  deprescribing: Clinical history and severity of the disease being studied (e.g., time since diabetes diagnosis). | 0 (0%) | 4 (7%) | 51 (91%) | 1 (2%) | Include |
| 17 | Investigators should consider including the following covariate in studies of deprescribing: Co-prescribed medications. | 1 (2%) | 5 (9%) | 49 (86%) | 2 (4%) | Include |
| 18 | Investigators should consider including the following covariate in studies of deprescribing: Comorbidities. | 0 (0%) | 3 (5%) | 53 (93%) | 1 (2%) | Include |
| 19 | Investigators should consider including the following covariate in studies of deprescribing: Global assessments of comorbidity, frailty, or mortality risk. | 3 (5%) | 4 (7%) | 47 (83%) | 3 (5%) | Include |
| 20 | Investigators should consider including the following covariate in studies of deprescribing: Healthcare provider characteristics. | 6 (11%) | 15 (26%) | 34 (60%) | 2 (4%) | Resurvey |
| 21 | Investigators should consider including the following covariate in studies of deprescribing: Family caregiver characteristics and involvement in care. | 5 (9%) | 47 (83%) | 2 (4%) | 3 (5%) | Resurvey |
| 22 | Investigators should consider including the following covariate in studies of deprescribing: Relevant policies or clinical guidelines in place during the study  period. | 5 (9%) | 8 (14%) | 38 (68%) | 5 (9%) | Resurvey |
| 23 | Investigators should consider including the following covariate in studies of deprescribing: Health system or facility characteristics, resources, and culture. | 5 (9%) | 10 (18%) | 34 (61%) | 7 (13%) | Resurvey |

Free-text items:

1. What is your current career stage?
2. In which country are you currently located?
3. Are there any topics or reporting areas you believe are important but were missed in this survey? If yes, please explain.

**Table S2a.** Distribution of Geographic Location of Round 1 Participants (N=61).

| **Country** | **N** | **Percent of Total** |
| --- | --- | --- |
| Australia | 3 | 5% |
| Canada | 2 | 3% |
| Chile | 1 | 2% |
| China | 1 | 2% |
| Denmark | 4 | 7% |
| France | 1 | 2% |
| Germany | 2 | 3% |
| Hong Kong | 1 | 2% |
| Ireland | 1 | 2% |
| Italy | 1 | 2% |
| Netherlands | 3 | 5% |
| Qatar | 1 | 2% |
| Republic of Korea | 2 | 3% |
| Romania | 1 | 2% |
| Slovenia | 2 | 3% |
| Sweden | 3 | 5% |
| Switzerland | 2 | 3% |
| Taiwan | 1 | 2% |
| UK | 7 | 11% |
| US | 22 | 36% |

**Table S2b.** Distribution of Career Stage of Round 1 Participants (N=66).

| **Country** | **N** | **Percentage of Total** |
| --- | --- | --- |
| Trainee (student, postdoc, etc.) | 24 | 36% |
| Early Career: 10 years or fewer from terminal degree | 16 | 24% |
| Mid-Career: 10-20 years from terminal degree | 15 | 22% |
| Senior: 20 or more years from terminal degree | 11 | 17% |

**Table S3.** Free text responses from Round 1 of the survey and actions taken.

| **Comment** | **Are there any topics or reporting areas you believe are important but were missed in this survey? If yes, please explain.** | **Action** |
| --- | --- | --- |
| 1 | Probably you can add potential deprescribing definitions details. | Disregard - Already Included |
| 2 | Cost, insurance switch, formulary | Discuss in manuscript to provide additional details/context |
| 3 | Re-starting or time to re-start the previously deprescribed medication. | New candidate recommendation |
| 4 | How to deal with relapse, one major risk of deprescribing? Should those patients be simply censored? | Disregard - duplicate concept as prior item |
| 5 | How tapering was defined in routinely collected data | Discuss in manuscript to provide additional details/context |
| 6 | Data source, indication, population (children deprescribe too), reason for deRx when available | Disregard - Out of Scope |
| 7 | Outcome (research question) selection | Disregard - Already Included |
| 8 | Which methods we should use to address every challenge | Disregard - Out of Scope |
| 9 | Methods used to define time zero in de-prescribing studies | Discuss in manuscript to provide additional details/context |
| 10 | The effects of exposure misclassification on effect estimate. | Discuss in manuscript to provide additional details/context |
| 11 | Critical emergency situations such as war, COVID 19 | Disregard - Out of Scope |
| 12 | Sensitivity analyses to address residual confoundings or uncertainty in estimates caused by study assumptions | New candidate recommendation |
| 13 | Patients who re-initiate prescribing. | Disregard - duplicate concept as prior item |
| 14 | Other health service use | Discuss in manuscript to provide additional details/context |
| 15 | Patient preferences | Discuss in manuscript to provide additional details/context |
| 16 | Health services use by participants, as an important proxy for health status | Disregard - Already Included |
| 17 | Focus on individuals medication across a time continuum, versus more broadly total medication burden that is intervened on periodically (e g. Quarterly) which is closer to actual practice. | Disregard - Out of Scope |
| 18 | Diversity by race of data sources subjects as most studies are in White population | Discuss in manuscript to provide additional details/context |
| 19 | Justification for the chosen study design, with consideration given to time related and other biases, unmeasured confounders etc | Discuss in manuscript to provide additional details/context |

**Table S4.** Survey items and responses (Round 2, n=25 total).

| **Item Number** | **Item** | **Not Important/Somewhat Unimportant (N, % of total responses for that item)** | **Neither Important nor Unimportant (N, % of total responses for that item)** | **Somewhat Important or Very Important (N, % of total responses for that item)** | **Result** |
| --- | --- | --- | --- | --- | --- |
| 1 | Investigators should consider including the following covariate in studies of deprescribing: Healthcare provider characteristics | 3 (12%) | 2 (8%) | 20 (80%) | Include |
| 2 | Investigators should consider including the following covariate in studies of deprescribing: Family caregiver characteristics and involvement in care | 5 (20%) | 4 (16%) | 16 (64%) | Exclude |
| 3 | Investigators should consider including the following covariate in studies of deprescribing: Relevant policies or clinical guidelines in place during the study period | 4 (16%) | 3 (12%) | 18 (72%) | Include |
| 4 | Investigators should consider including the following covariate in studies of deprescribing: Health system or facility characteristics, resources, and culture | 3 (12%) | 8 (32%) | 14 (56%) | Exclude |
| 5 | When applicable, investigators should avoid inducing immortal time (e.g., by misclassifying follow-up time before meeting the minimum medication-free interval into the "deprescribed" group or excluding it from analysis). | 1 (4%) | 2 (8%) | 22 (88%) | Include |
| 6 | The definition of deprescribing should include consideration of time-varying exposure status (e.g., restart of therapy after deprescribing, therapeutic substitution), through methods such as intention-to-treat analyses, time-varying exposure measures, or censoring. | 0 (0%) | 3 (12%) | 22 (88%) | Include |
| 7 | Investigators should consider conducting sensitivity analyses regarding residual confounding, in particular confounding by indication. | 1 (4%) | 1 (4%) | 23 (92%) | Include |

**Supplemental Table 5.** Reporting and Methods checklist for Observational Studies on the Effects of Deprescribing (REMROSE-D).

| **Section** | **Item Supplemental Table 5**. | **Reported or described on page number(s)** | **If not reported or applicable, explanation** |
| --- | --- | --- | --- |
| **General Reporting and Study Design** | 1. Follow a set of accepted general reporting guidelines for the specific type of observational study leveraged. |  |  |
|  | 2. Present a study diagram that clearly identifies exposure, covariate, outcome, and eligibility assessment periods and the index date/time zero, as applicable. |  |  |
| **Deprescribing as an Exposure** | 3. Provide a detailed description of how exposure was assessed, including the data source, data transformations, how the last exposed date was determined, and any limitations of the data source to capture the most relevant form of deprescribing, as applicable. |  |  |
|  | 4. Justify the decision to define deprescribing as completely stopping the medication, tapering the medication, or another definition in the context of the specific research question or clinical scenario. |  |  |
|  | 5. Discuss whether the definition of deprescribing was informed by clinical perspectives, including common prescribing practices and clinical practice guidelines. |  |  |
|  | 6. Discuss whether the definition of deprescribing was informed by pharmacokinetic considerations, including the duration of potential effects of the drug on outcomes after discontinuation. |  |  |
|  | 7. Discuss design and analytic choices implemented with exposure changes over time, including as-treated vs. intention-to-treat analyses, censoring, and/or time-varying exposure methods. |  |  |
|  | 8. Consider using similar definitions for deprescribing as prior studies, at least in secondary analyses, to enhance comparability of results. |  |  |
|  | 9. When applicable, avoid inducing immortal time (e.g., by misclassifying follow-up time before meeting the minimum medication-free interval into the "deprescribed" group or excluding it from analysis). |  |  |
|  | 10. Test the potential influence of the definition for deprescribing on study results through sensitivity analyses |  |  |
| **Assessment and Outcomes** | 11. Discuss the time by which you would expect outcomes to occur relative to deprescribing (i.e., short-term vs. long-term outcomes). |  |  |
|  | 12. Include outcomes related to the negative potential consequences of deprescribing when possible. |  |  |
|  | 13. Include outcomes related to the potential benefits of deprescribing when possible. |  |  |
| **Considerations for Confounding** | 14. Discuss the comparator group in terms of potential for confounding, especially as it relates to differences between people who discontinue a medication vs. continue treatment. |  |  |
|  | 15. Select covariates based on the clinical and practical relevance to the selected drug class as permissible by the data source. |  |  |
|  | 16. Conduct sensitivity analyses regarding residual confounding, in particular confounding by indication. |  |  |
|  | Consider the following covariates: |  |  |
|  | 17. Sociodemographic information, including age, race/ethnicity, sex, and geography. |  |  |
|  | 18. Clinical history and severity of the disease being studied |  |  |
|  | 19. Co-prescribed medications |  |  |
|  | 20. Comorbidities |  |  |
|  | 21. Prognosis and global assessments of comorbidity, frailty, or mortality risk |  |  |
|  | 22. Healthcare provider characteristics |  |  |
|  | 23. Relevant policies or clinical guidelines in place during the study period |  |  |

**Supplemental Material 1.** Slides from presentation delivered before Round 1 survey.

*Citation: Hayes KN, Niznik JD, Moga DC, Maciejewski M, Moriarty F, Johnson CM. Methodological guidance for real world evidence to assess the effects of deprescribing. Symposium and Workshop, International Society for Pharmacoepidemiology Annual Meeting, Aug 2024, Berlin, Germany.*

*Full paper can be found at:* ***Hayes KN,*** *Niznik JD, Gnjidic D, et al. Evaluation of real-world evidence to assess health outcomes related to deprescribing medications in older adults: an International Society for Pharmacoepidemiology-endorsed systematic review of methodology.* ***Am J Epidemiol.*** *2025;914(8): 2431–2439. PMID: 39572376. PMCID: PMC12342872.*

*
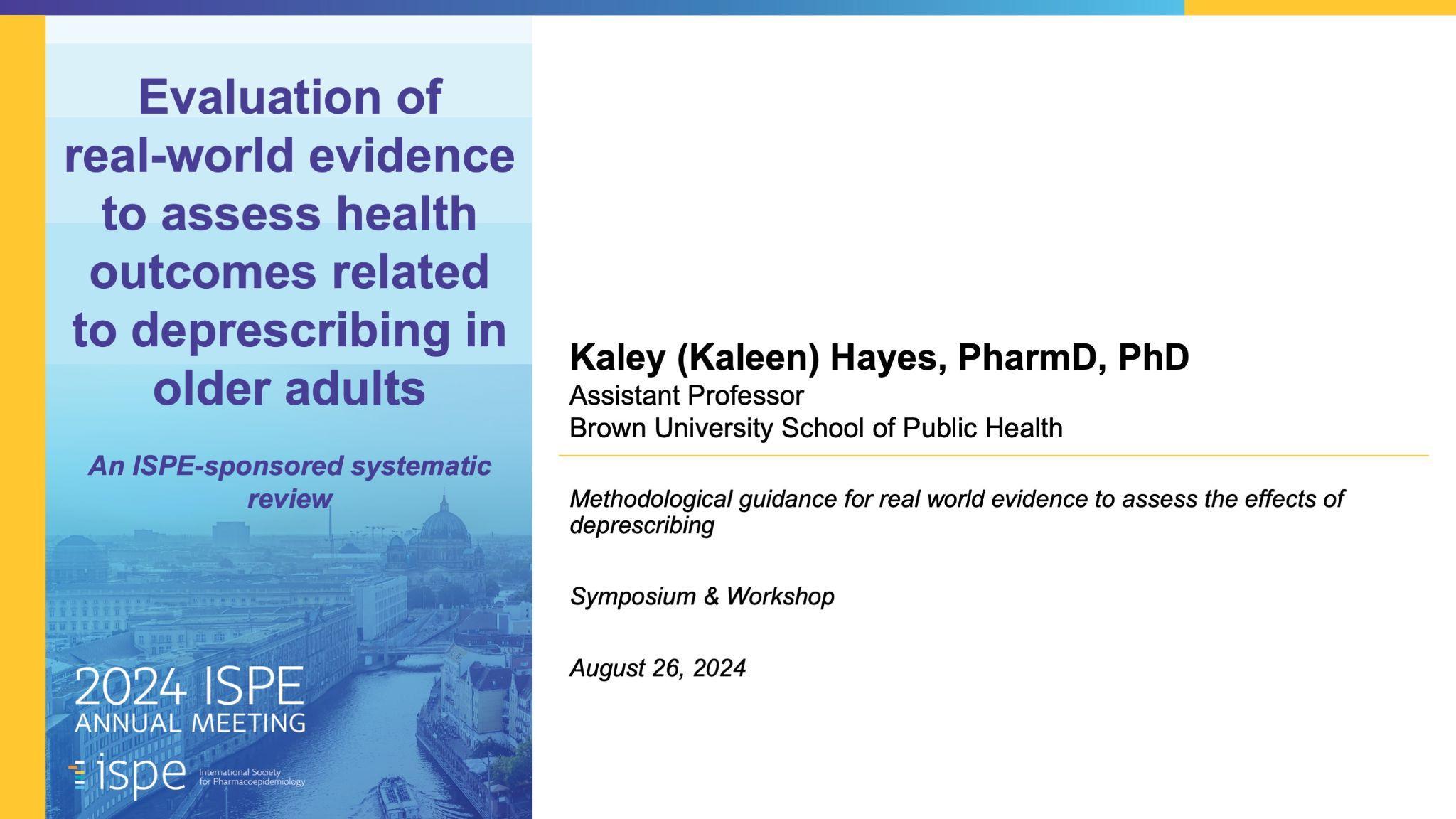
*


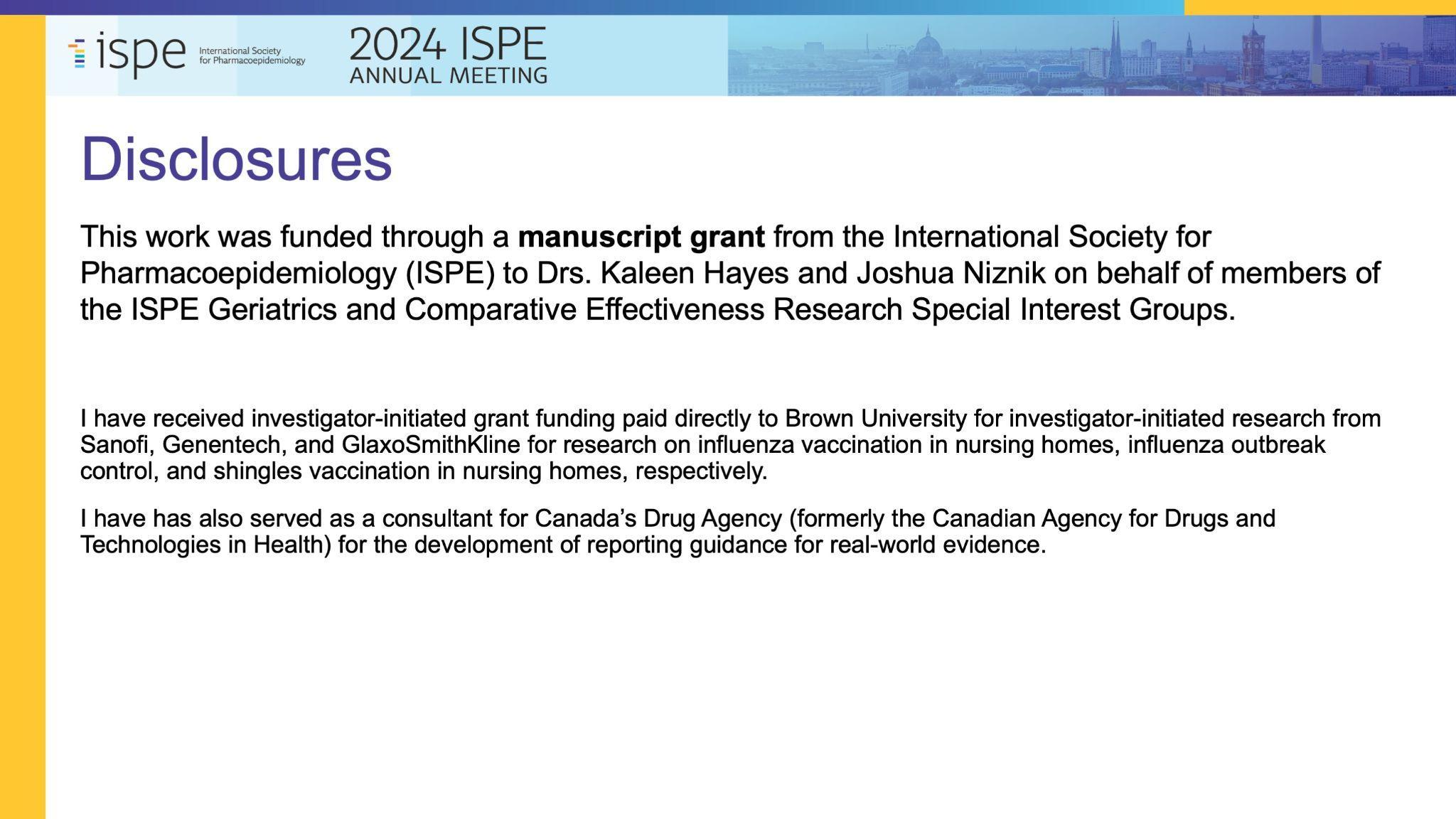


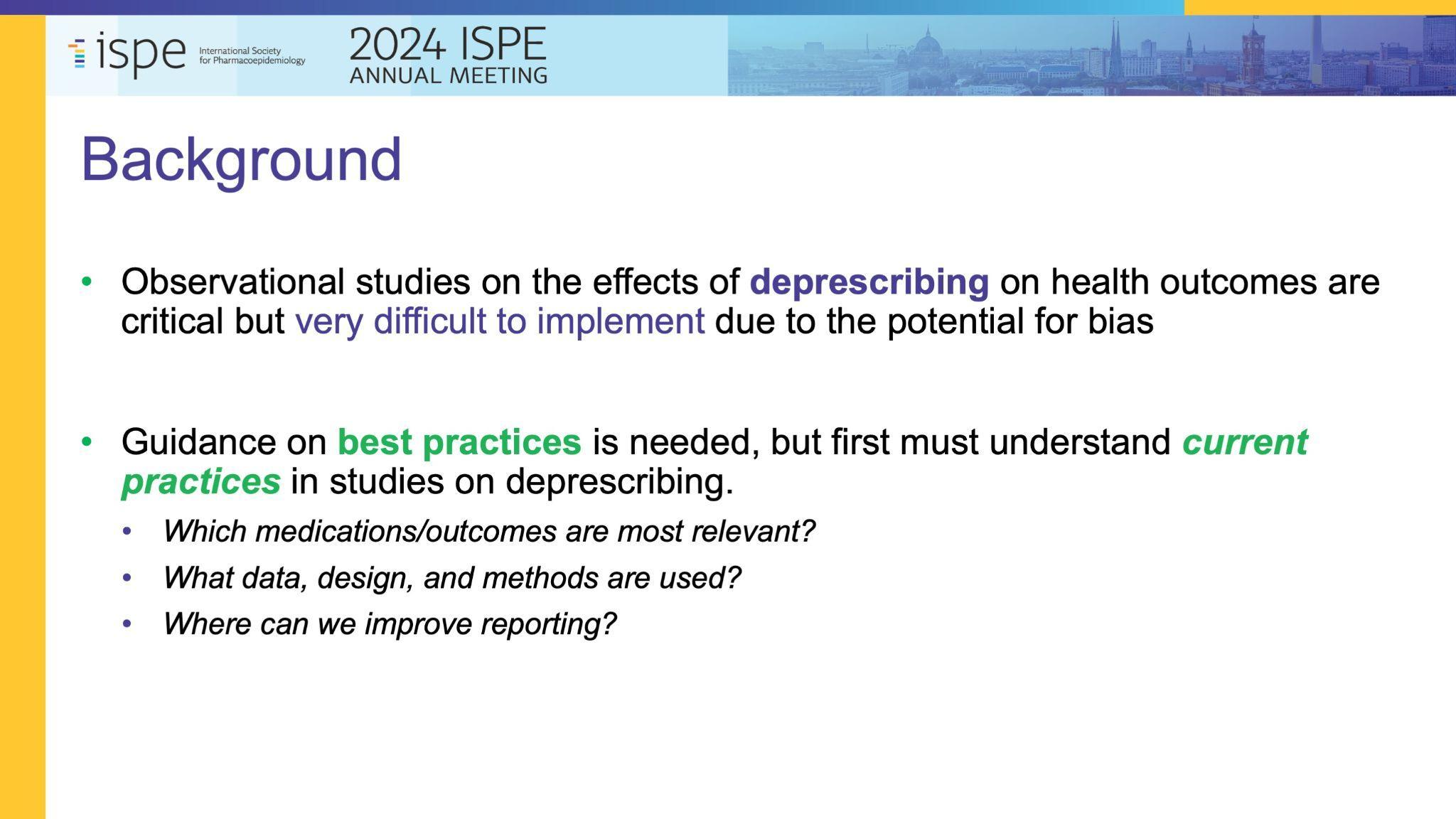


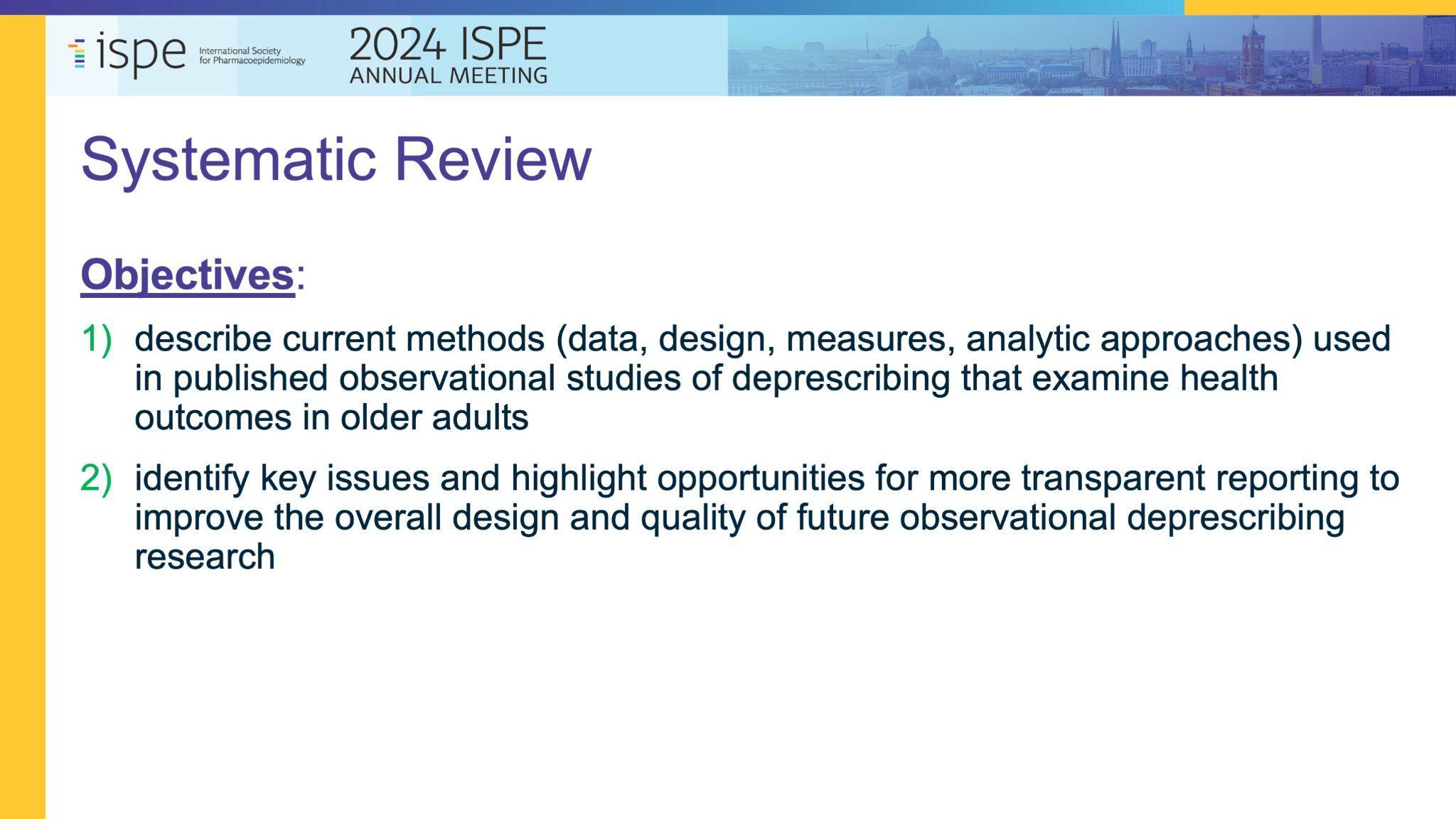

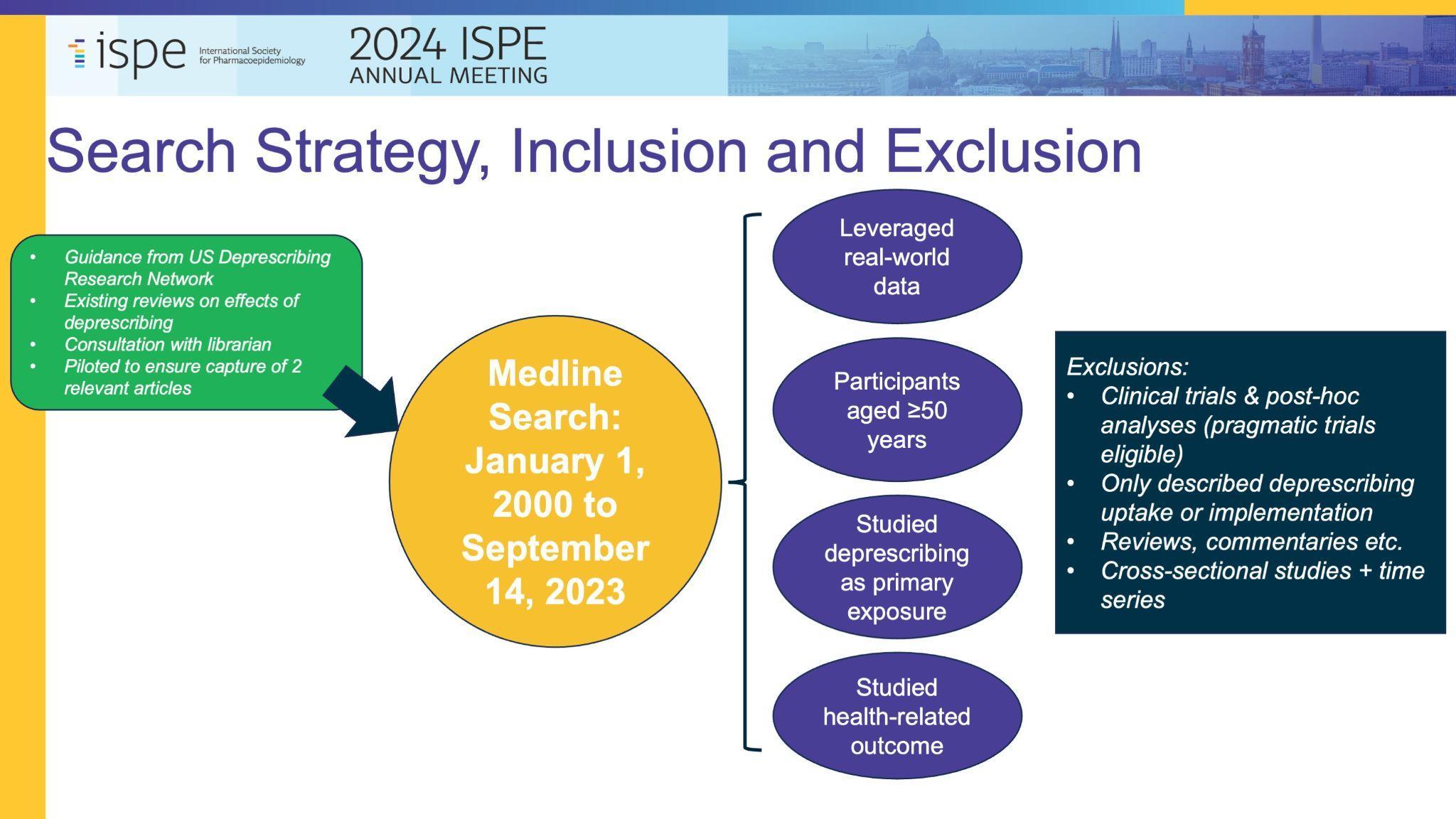

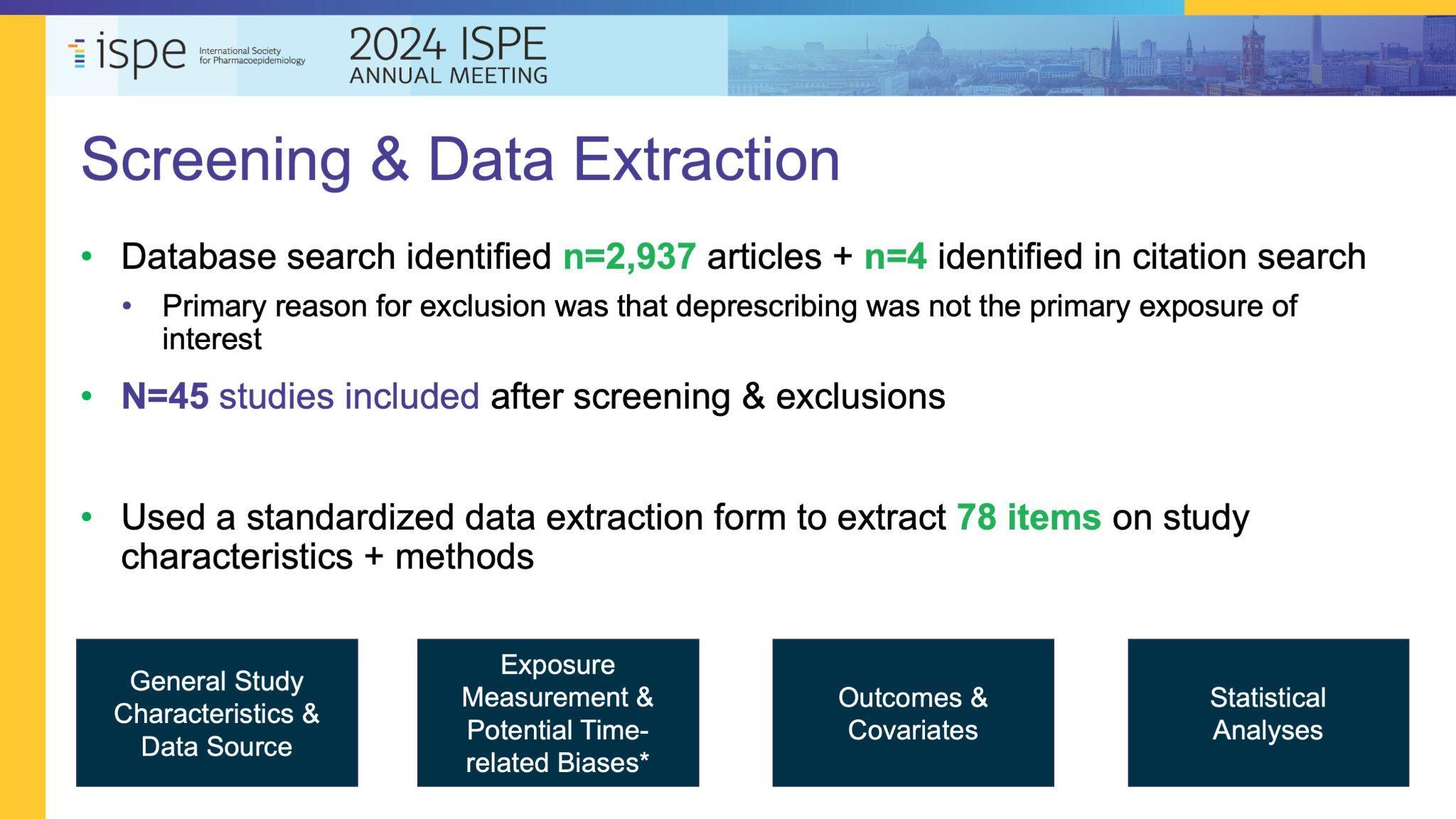

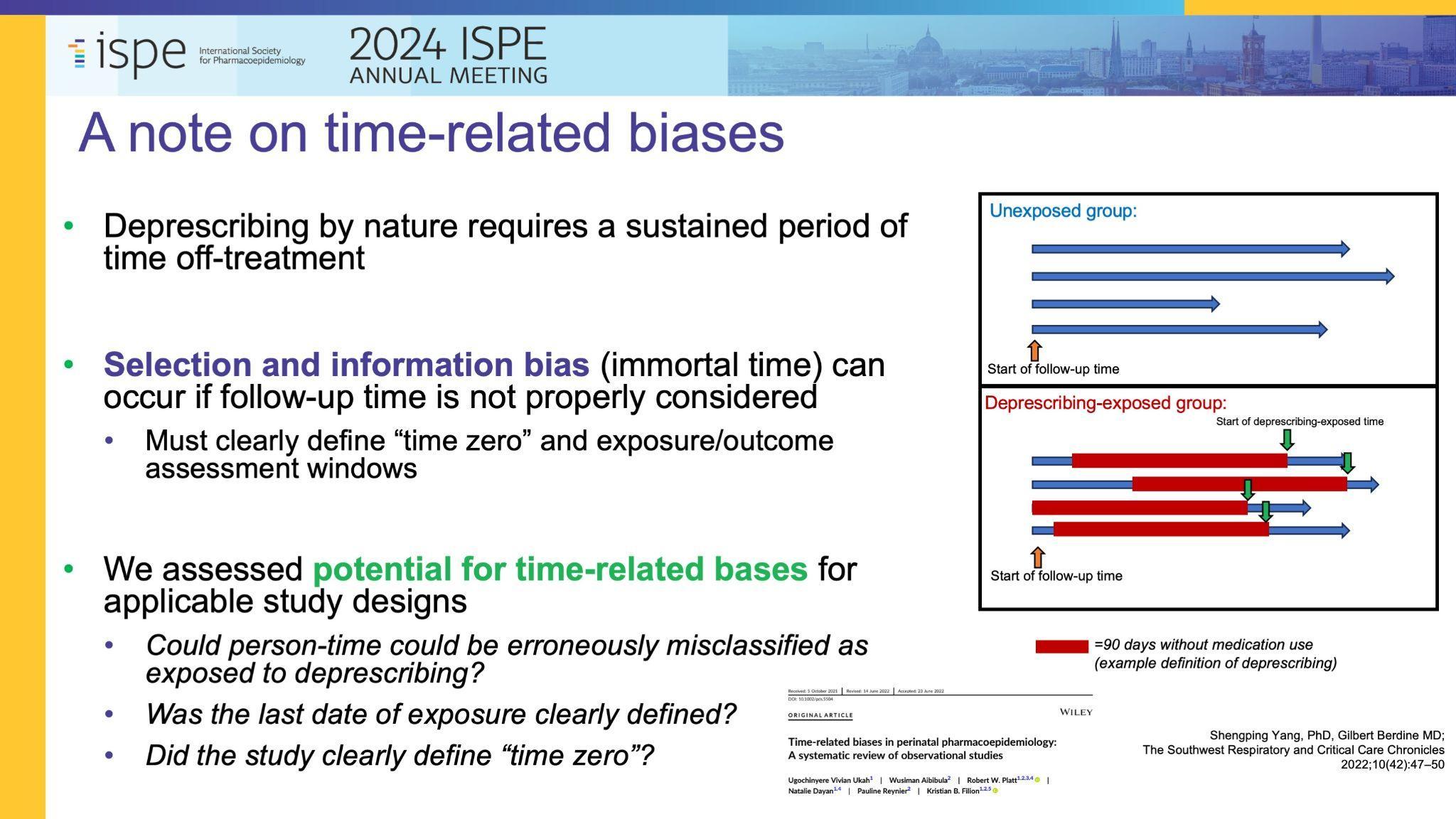

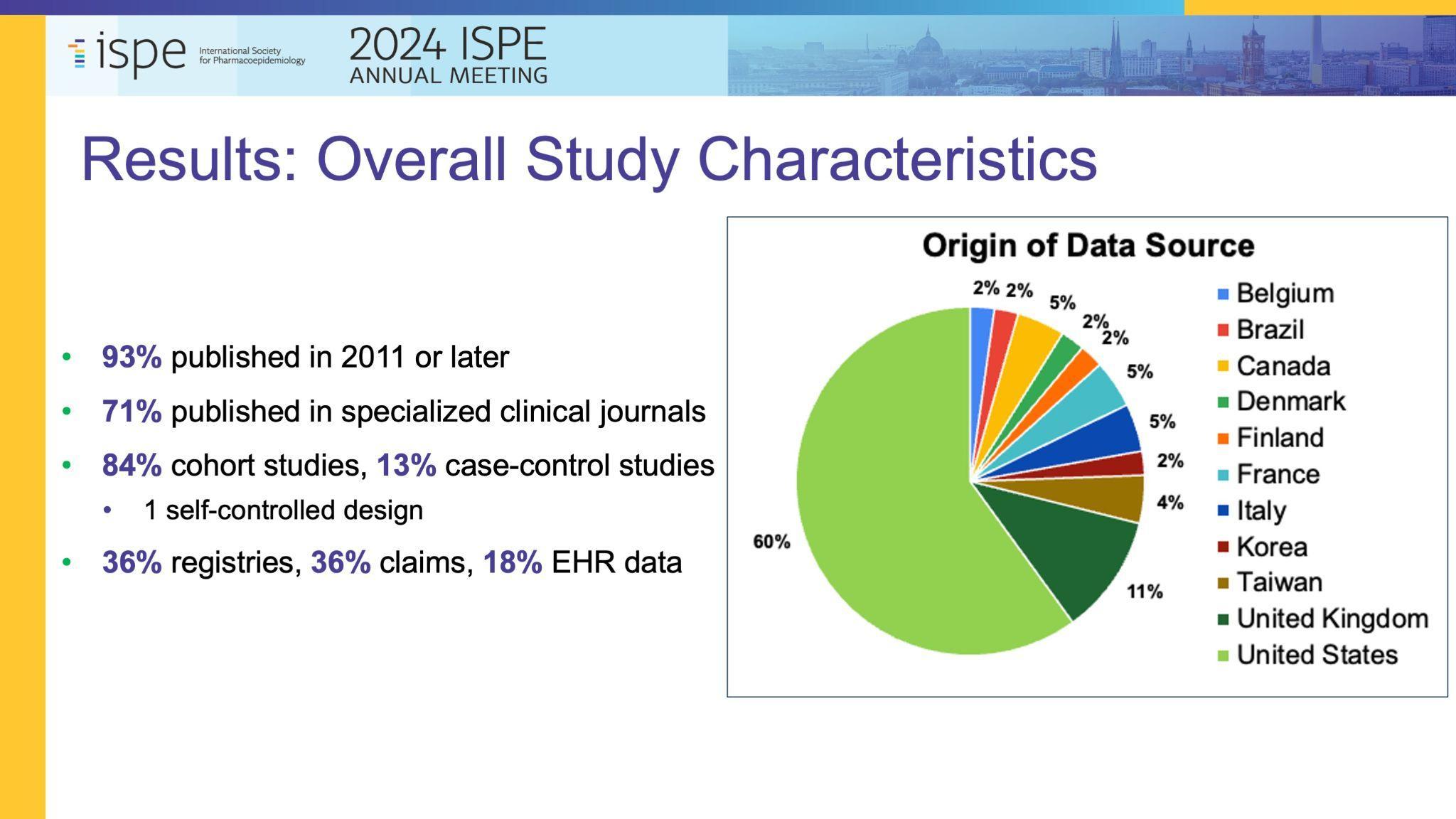

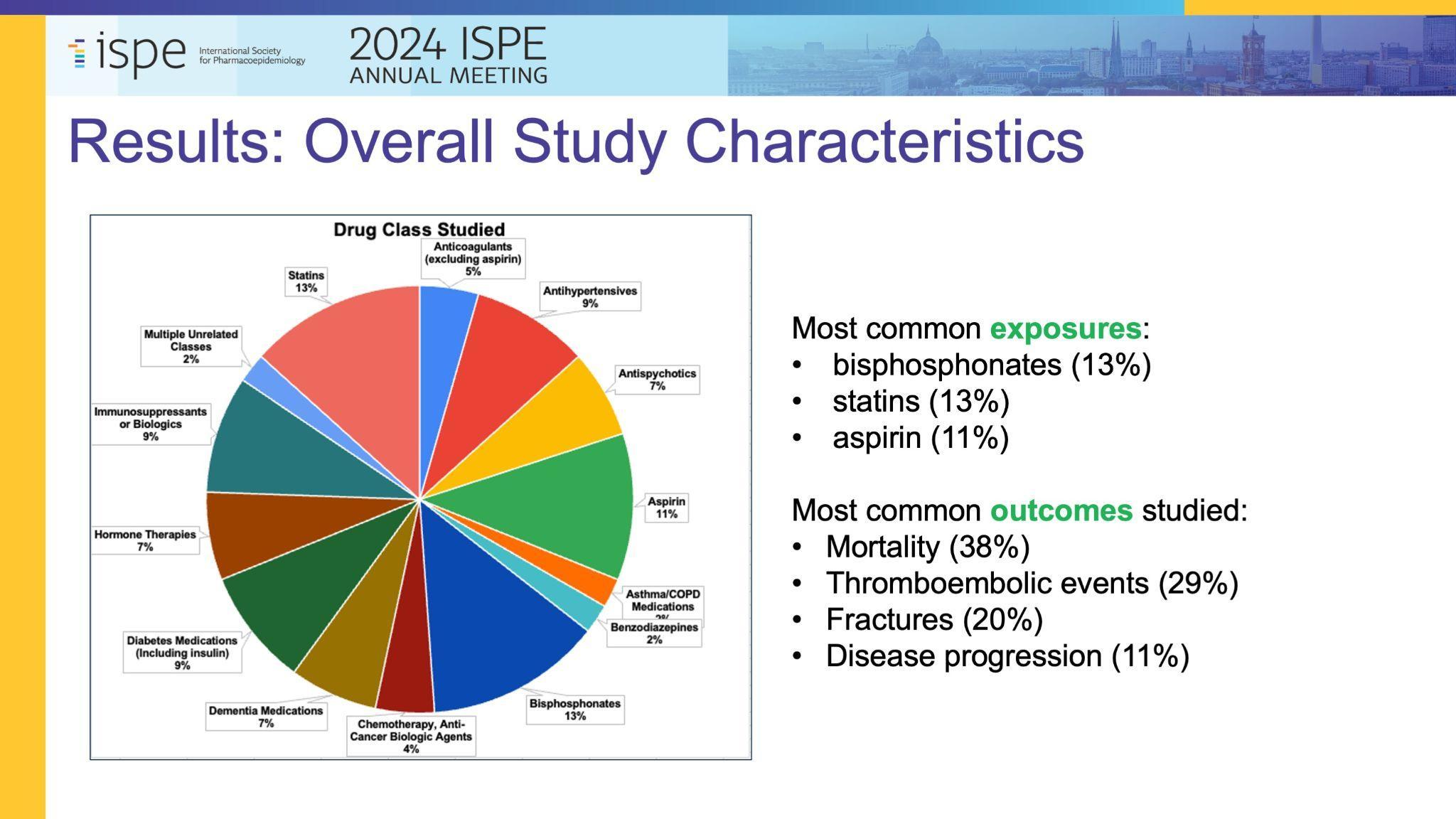

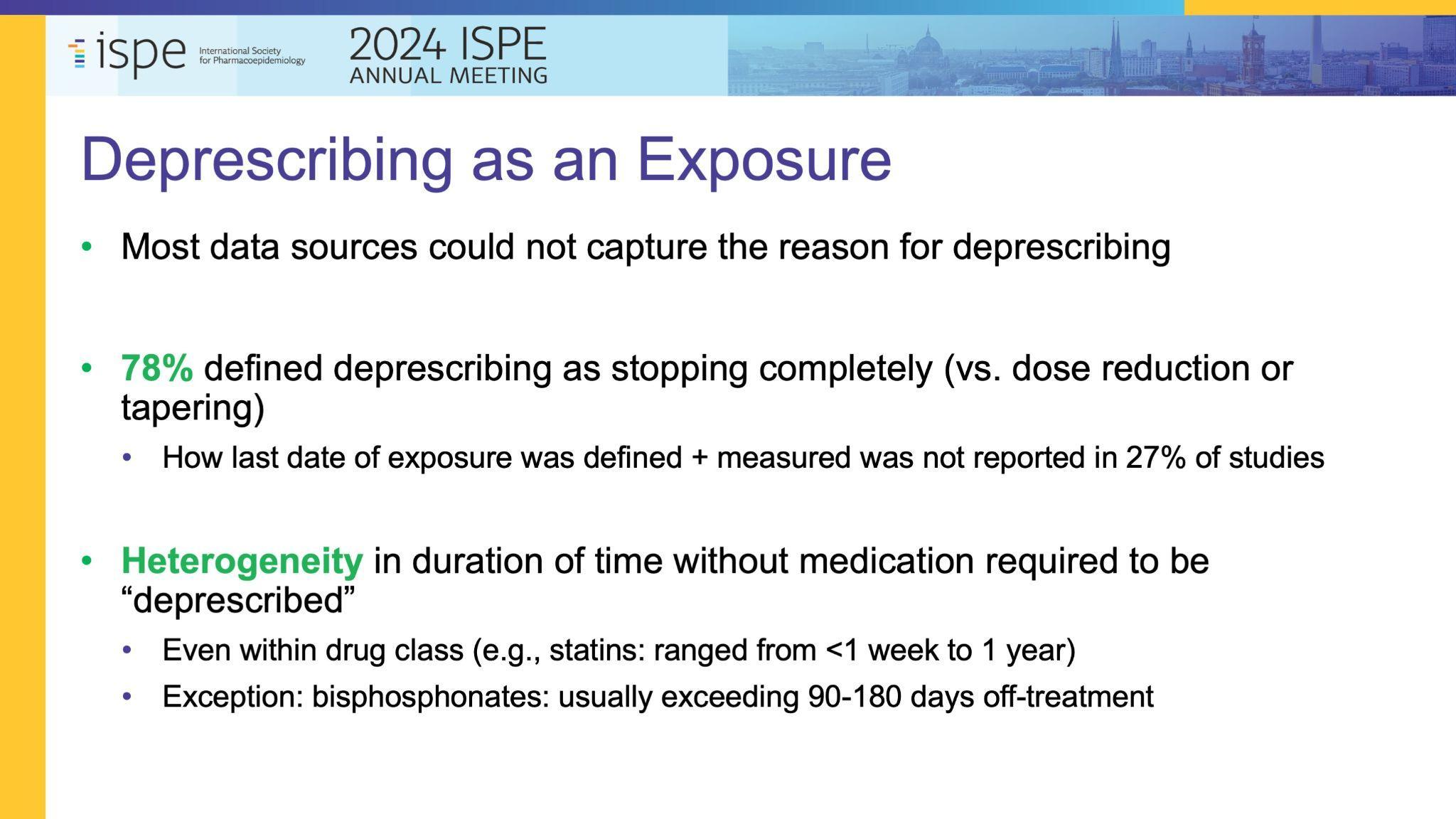

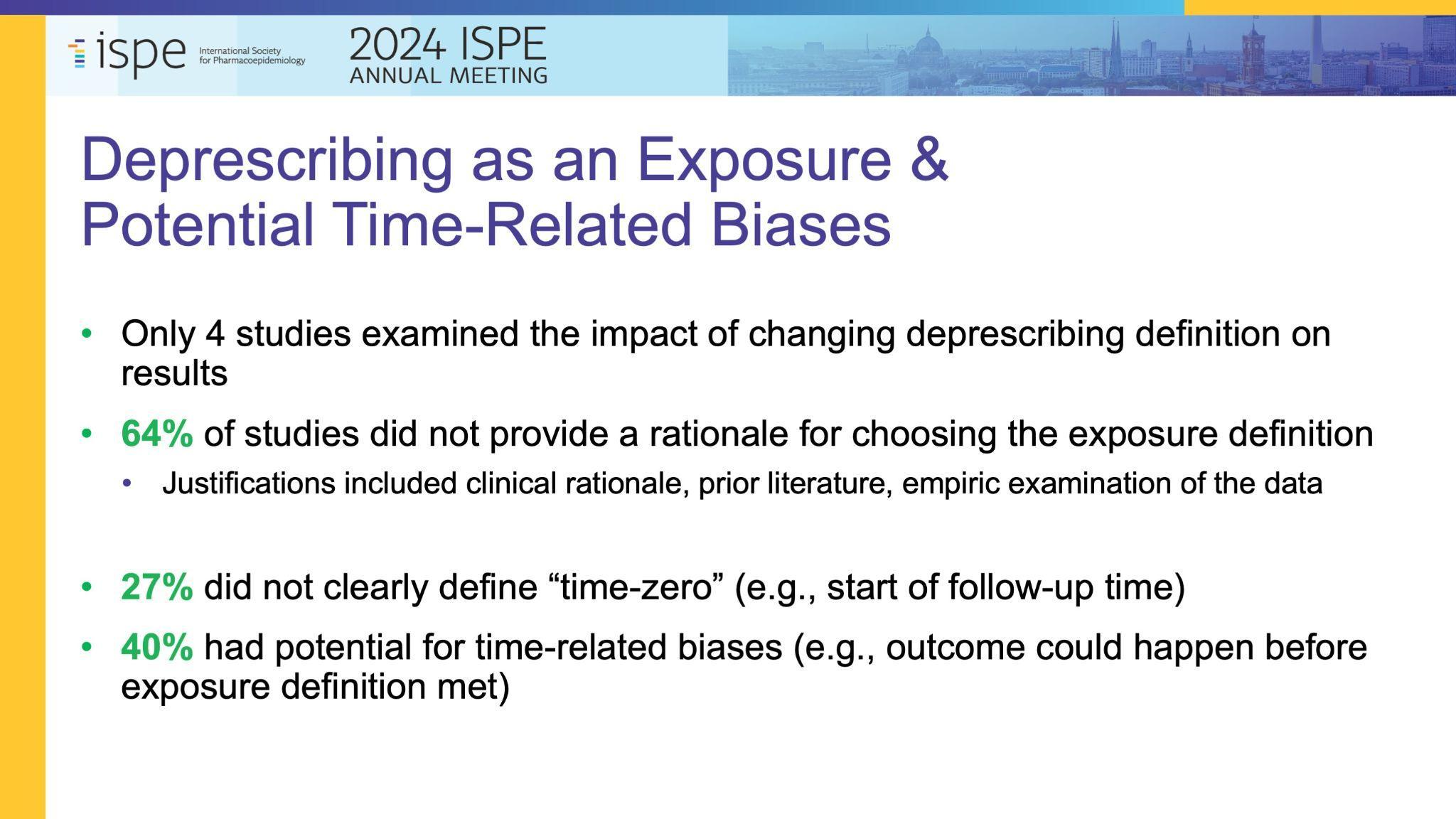

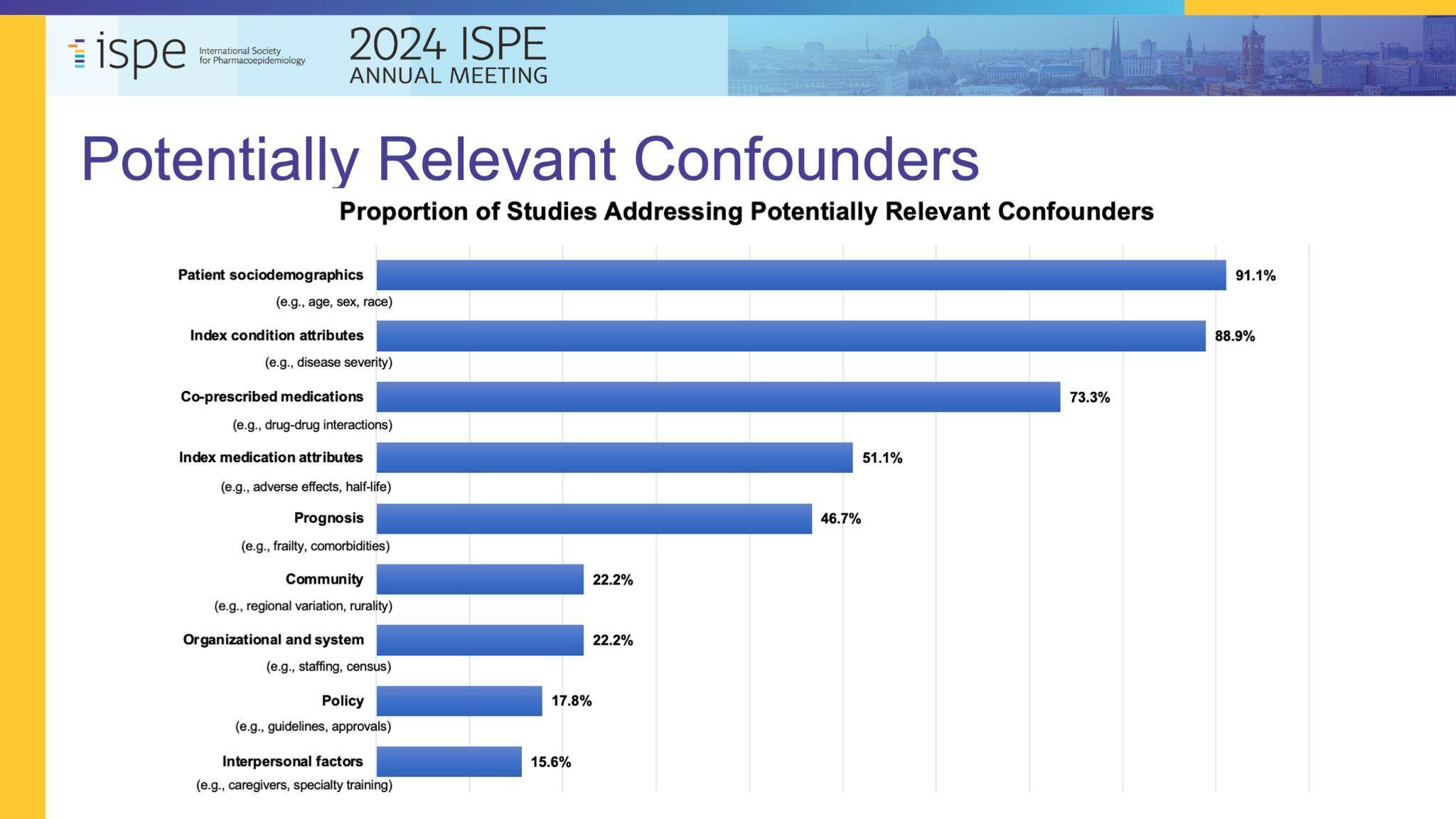

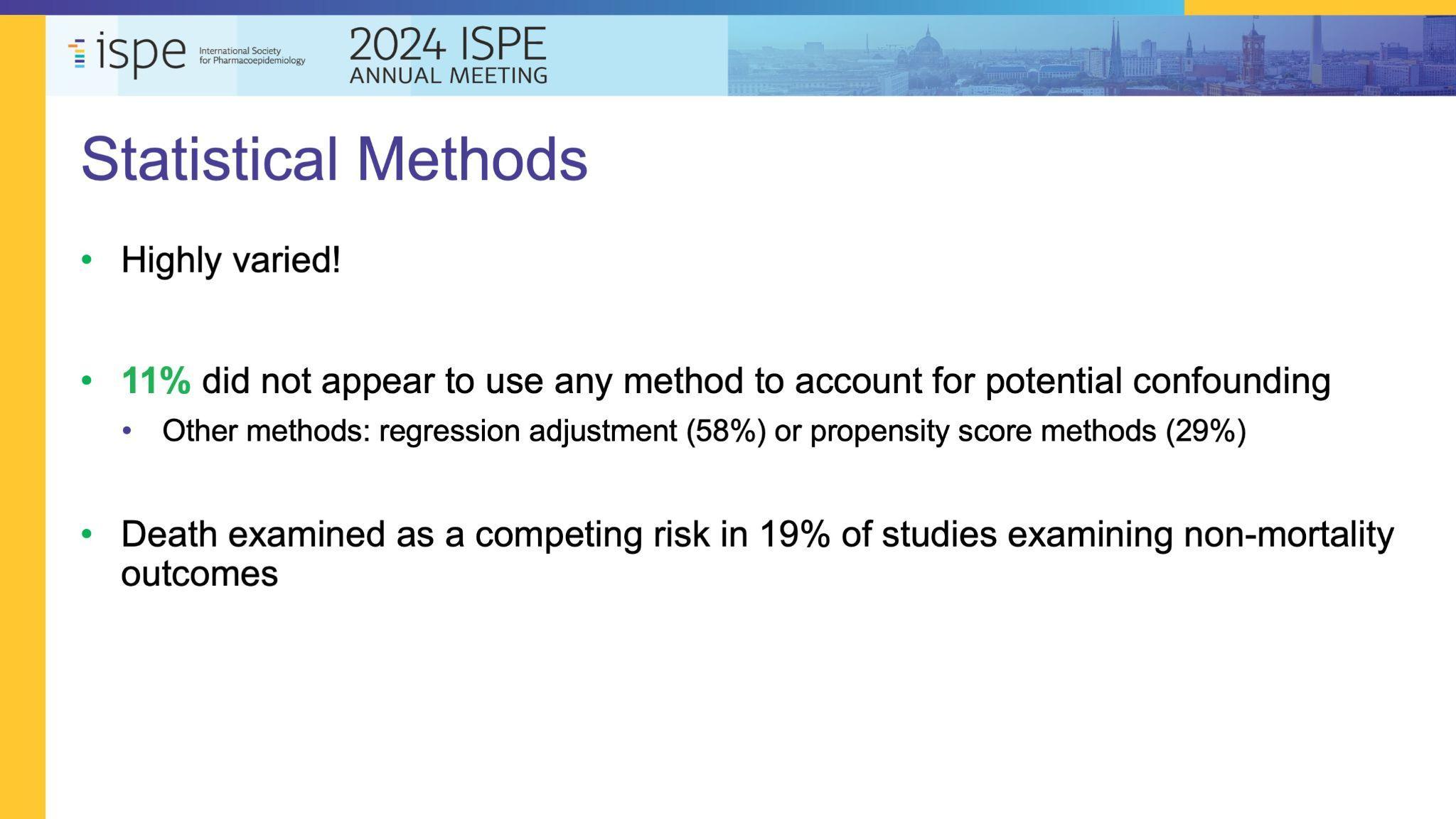

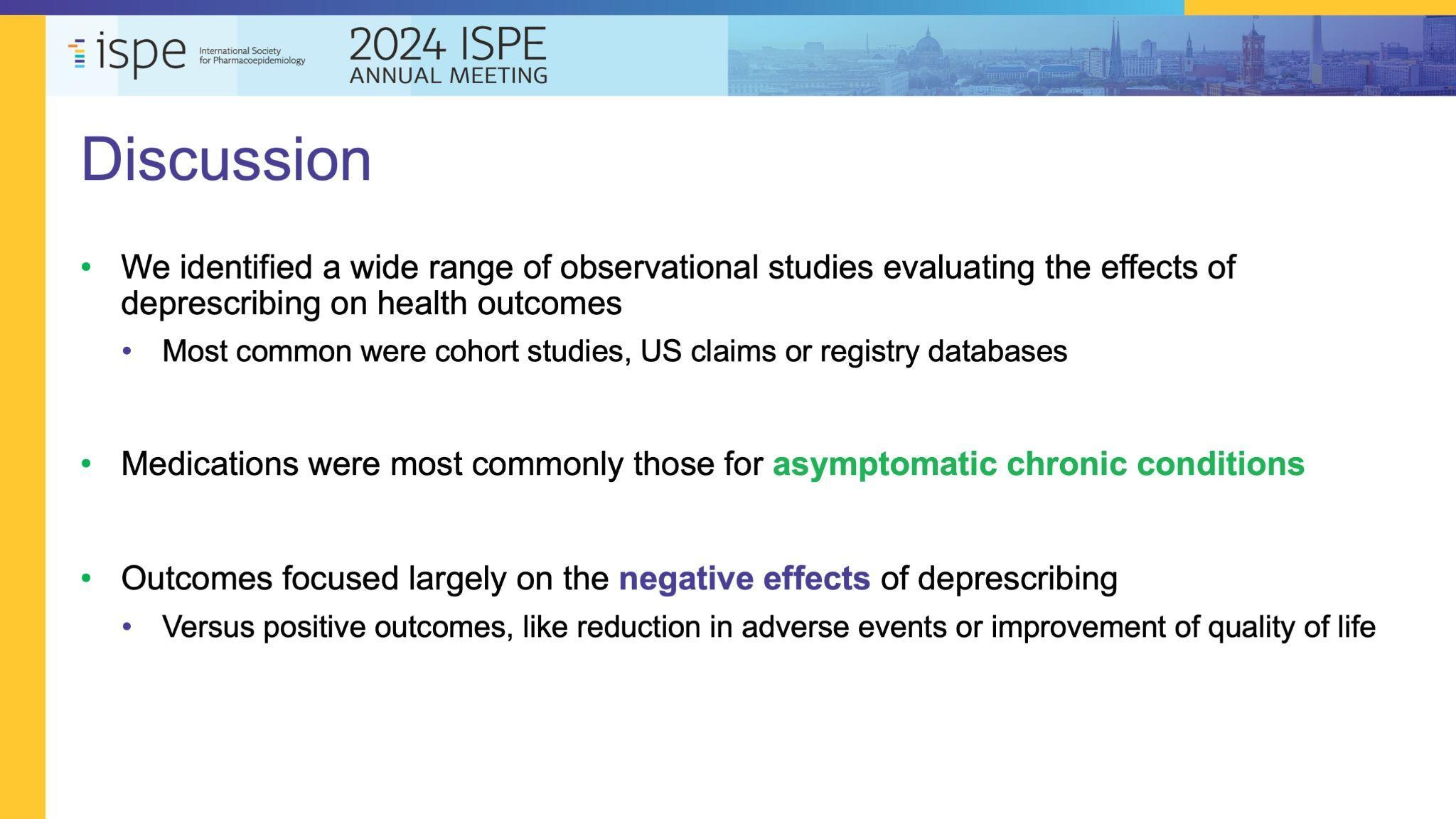

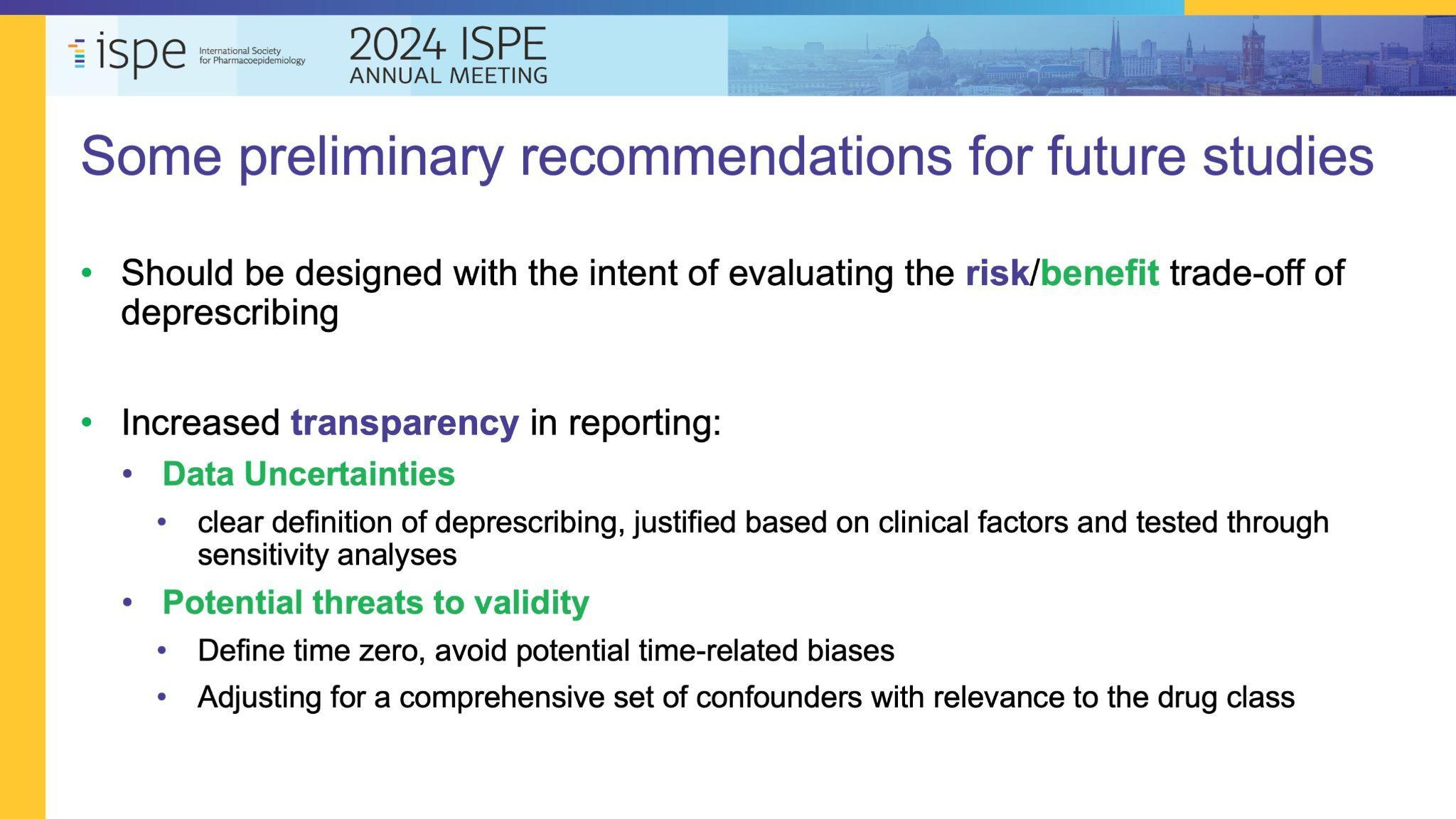

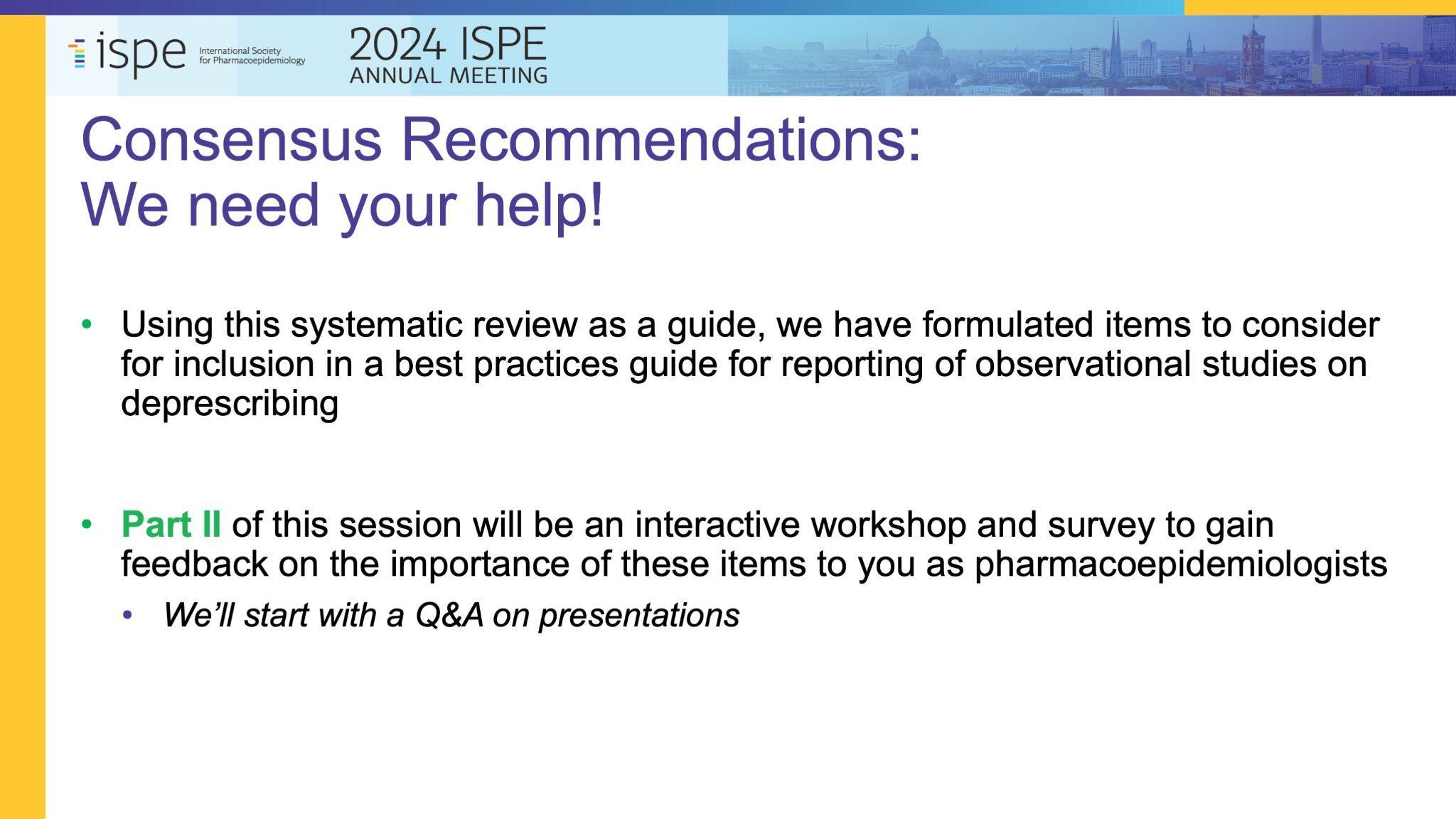

Supplement: Supplementary file 1 — Data S1: pds70255‐sup‐0001‐Supinfo.docx. [file PDS-34-e70255-s001.docx]
